# Development of a Liposome-Based Serological Assay for SARS-CoV‑2 Variants with Special Emphasis on Coupling Chemistries Required to Maintain Protein Antigenicity

**DOI:** 10.1021/acs.analchem.5c02526

**Published:** 2025-09-05

**Authors:** Simon Streif, Patrick Neckermann, Kilian Hoecherl, Christina Reiner, Sebastian Einhauser, Johannes Konrad, Miriam Breunig, Ralf Wagner, Antje J. Baeumner

**Affiliations:** † Institute of Analytical Chemistry, Chemo- and Biosensors, 9147 University of Regensburg, Universitaetsstr. 31, Regensburg 93053, Germany; ‡ Institute of Medical Microbiology & Hygiene, Molecular Microbiology (Virology), 210421 University of Regensburg, Universitaetsstr. 31, Regensburg 93053, Germany; § Department of Pharmaceutical Technology, University of Regensburg, Universitaetsstr. 31, Regensburg 93053, Germany; ∥ Institute of Clinical Microbiology and Hygiene, University Hospital Regensburg, Franz-Josef-Strauss-Allee 11, Regensburg 93053, Germany

## Abstract

The conjugation of proteins to the outer membranes of liposomes is a standard procedure used in bioanalytical and drug delivery approaches. Herein, we describe the development of a liposome-based surrogate assay for the quantification of SARS-CoV-2 neutralizing antibodies. Taking into consideration differences in amino acid sequences within the receptor-binding domain (RBD) of SARS-CoV-2 Spike proteins derived from five selected variants of concern (VoC), we studied the impact of coupling chemistries on physicochemical properties and antigenicity. Naturally occurring lysine residues were used for standard EDC/NHS chemistry, while an N-terminal Cys-tag and a C-terminal Avi-tag were genetically added to the proteins for site-directed immobilization. Despite only minor differences regarding the number, positioning, and sequence context of lysine residues within the different RBD variants, those differences led to a dramatic change in their functionality after EDC/NHS coupling. In contrast, site-specific biotinylation of the proteins alongside targeted immobilization on streptavidin- or neutravidin-modified liposomes resulted in restored functionality and enhanced storage stability across all variants. The developed adaptable liposome-based test showed excellent correlation with an established pseudovirus neutralization test and could identify variations in neutralization patterns of Alpha/Delta and Omicron variants in patient sera. The study highlights the benefits of using neutravidin-liposomes for site-directed protein immobilization with independence from the proteins’ amino acid sequences, enhanced storage stability, and applicability to various biotinylation strategies, serving as a versatile platform technology that can also be applied to the coupling of other proteins or peptides used for diagnostic purposes.

## Graphical abstract

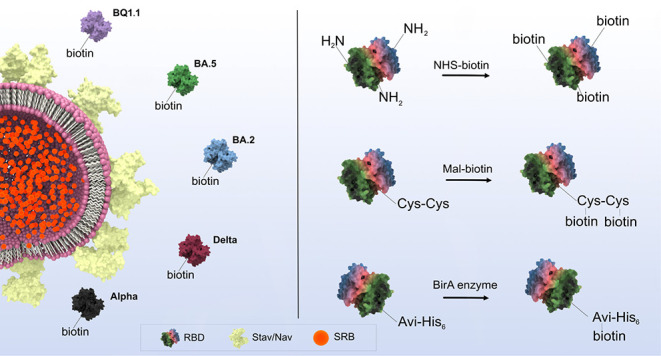

## Introduction

Liposomes, self-assembled spherical vesicles consisting of a lipid bilayer and an aqueous cavity, have been widely applied in both drug delivery and bioanalytical tests. The large inner cavities allow for encapsulation of different drugs or markers, e.g., *m*-carboxyluminol,^1^ redox markers such as ferri/ferro hexacyanide^2^ and Ru­(bpy)_3_^2+^,^3^ fluorophores,^4^ or even enzymes^5,6^ and hence easily support chemiluminescent, electrochemical, or optical multianalyte approaches. Liposomes are typically composed of phospholipids, with the hydrophobic tail lengths influencing the curvature and thus size,^7^ and sterols, such as cholesterol, reducing the permeability of solutes and increasing rigidity and dispersion stability.^8,9^ The use of phospholipids with functionalized headgroups, e.g., amine-, carboxy-, and thiol-groups, enables fine-tuning of the surface charge as well as modification of liposomes with other molecules. Besides small molecules (e.g., biotin and fluorescein) and polymers, such as poly­(ethylene glycol), they are often modified with proteins to facilitate specific interaction with analytes.^4,10–13^ The addition of EDC and sulfo-NHS to COOH-liposomes is commonly used to generate an amine-reactive sulfo-NHS-ester that subsequently reacts with NH_2_-groups of the protein. This can be either the α-amino group of the N-terminal amino acid or the ε-amino group of lysines.^14^ This approach therefore results in random orientation of lysine-containing proteins on the liposomal surface, further influenced by the pH value, with the α-amino group being favored at pH 7 and the reactivity of the ε-amino group being enhanced at pH 8.^15^ Alternative modification strategies include maleimide coupling to thiols or coupling of azide to alkyne groups using click chemistry. These require the incorporation of tags if site-directed coupling is desired, e.g., cysteine-tags for the former, while noncanonical amino acids, e.g., azido-homoalanine,^16^ are required for the latter. Otherwise, thiol-, azide-, or alkyne-NHS-esters can be used to randomly modify the protein via its amine groups.^4,11^ Typically, the modification strategy is chosen case-by-case based on the chemistry necessary for coupling the functional group of a liposome to a (tagged-) protein. However, in an ideal scenario one would have a ready-to-use liposome that always carries the same functional group but can be used in a multitude of different assays simply by changing the (tagged) protein, without the need for further optimization of additional coupling steps.

The global spread of SARS-CoV-2, combined with the rapid development and authorization of protective vaccines based on the viral Spike proteins—the major surface protein of SARS-CoV-2—resulted in strong selection pressure against the Spike protein.^17,18^ Neutralizing antibodies targeting the receptor-binding domain (RBD), a 27 kDa domain of the trimeric Spike protein responsible for binding to the cellular angiotensin-converting enzyme 2 (ACE2) receptor, were quickly identified as a potential correlate of protection (CoP).^19–21^ Over time, RBD underwent extensive mutations to escape control by neutralizing antibodies, leading to the emergence of different variants of concern (VOC) with continuously shifting predominance.^18,22^ The first worldwide prevalent VOC was Alpha, occurring in the end of 2020, followed by the lineage Delta since mid of 2021. Delta was replaced by different sublineages of Omicron since beginning of 2022, ranging from BA.1, BA.2, BA.5, BQ1.1 toward the most recent strains of KP.3 sublineage.^23^ All strains of the Omicron sublineage exhibited a significant number of mutations within the RBD that altered antibody binding. Furthermore, these amino acid variations changed the physicochemical properties, particularly by increasing the pI, thereby making the RBDs more electropositive.^24^ Serological tests for SARS-CoV-2 immunity should ideally be adaptable to emerging VOCs. However, commercial binding antibody tests, such as the Elecsys anti-SARS-CoV-2 S (Roche) and cPass SARS-CoV-2 Neutralization Antibody Detection Kit (GenScript), still rely on the prototypic RBD.

In this article, different conjugation strategies for the modification of liposomes with proteins were investigated to provide a versatile platform technology that can easily be adapted to other analytes. The receptor-binding domain of SARS-CoV-2 (RBD) was chosen as a model protein due to its global importance, and five different variants were investigated (Alpha, Delta, BA.2, BA.5, and BQ1.1). Nondirected modification using standard coupling chemistries was compared to a site-directed approach using streptavidin-liposomes and RBD biotinylated via N-terminal Cys- or C-terminal Avi-tags. Finally, the optimal conjugation strategy was used to facilitate the quantification of neutralizing antibodies in patient serum samples for each variant.

## Experimental Section

### Chemicals and Consumables

All chemicals were of analytical reagent grade. Bovine serum albumin fraction V (BSA), cholesterol from sheep wool (C8667, ≥99%), human serum albumin (HSA), *N*-hydroxysulfosuccinimide sodium salt (sulfo-NHS, purity ≥98%), NHS-biotin (≥90%), Sephadex-G50 and G-100, skim milk powder (SMP), streptavidin from *Streptomyces avidinii*, Whatman Nucleopore Track-Etched membranes (1.0, 0.4, and 0.2 μm diameter), ethanol, acetic acid, Amicon centrifugal filters with a 10 kDa cutoff, and Tween 20 were purchased from Sigma-Aldrich/Merck (Darmstadt, Germany); 1,2-dipalmitoyl-*sn*-glycero-3-phosphoethanolamine-*N*-(glutaryl) (sodium salt) (*N*-glutaryl-DPPE) from NOF America Corporation (NY, USA); the remaining phospholipids, 1,2-dipalmitoyl-*sn*-glycero-3-phosphocholine (DPPC), 1,2-dipalmitoyl-*sn*-glycero-3-phospho-(1’-rac-glycerol) (sodium salt) (DPPG), and the extruder set were purchased from Avanti Polar Lipids (Alabaster, AL, USA). Sulforhodamine B (SRB) (S1307), (1-Ethyl-3-(3-(dimethylamino)­propyl) carbodiimide hydrochloride) (EDC) (PG82079), neutralizing SARS-CoV-2 Spike Protein (RBD) Polyclonal Antibodies (PA5-114451), neutravidin, and black high-binding 96-well microplates (Nunc MaxiSorp) were purchased from Thermo Fisher Scientific (Waltham, MA, USA); n-Octyl-β-d-glucopyranoside (OG) (≥98%, CN23), 2-(*N*-morpholino)-ethanesulfonic acid (MES) (≥99%, 4259), *N*-2-hydroxyethylpiperazine-*N*′-2-ethanesulfonic acid (HEPES) (≥99.5%, HN78), sucrose, sodium azide, Tris­(2-carboxyethyl)­phosphine (TCEP), imidazole (Cat. No. 2C4N.3), sodium chloride, and dialysis membrane Spectra/Por© 4 (MWCO: 12–14 kDa) (2718.1) were purchased from Carl Roth (Karlsruhe, Germany). Clear streptavidin-coated 96-well microplates (KaiSA96) were purchased from Uniogen (Turku, Finland). Phosphorus standard was obtained from Bernd Kraft GmbH (Den Haag, Netherlands). Chloroform, methanol, and Spectra-Por Float-A-Lyzer G2 (1 mL, MWCO: 1000 kDa) were purchased from Fisher Scientific (Hampton, NH, USA). *N*-succinimidyl 3-maleimidopropionate (Mal-NHS), Biotin-PEG_2_-amine were purchased from TCI (Eschborn, Germany), while bifunctional 3 kDa Maleimide-biotin-PEG was purchased from Rapp Polymere (Tuebingen, Germany). Coomassie R-250 was purchased from AppliChem (Darmstadt, Germany).

### Buffer Compositions

HEPES sucrose saline (HSS) buffer contained 200 mM sucrose, 200 mM NaCl, 10 mM HEPES, and 0.01 wt % NaN_3_, pH 7.5. PBS buffer contained 137 mM NaCl, 2.7 mM KCl, 10 mM Na_2_HPO_4_, and 1.8 mM KH_2_PO_4_, pH 7.4. PBS-T contained 0.1 wt % Tween 20 in PBS. MES buffer contained 50 mM MES, 200 mM sucrose, and 200 mM NaCl, pH 5.5.

### Cell Lines and Culture Conditions

Expi293 suspension cells (Thermo Fisher Scientific, Waltham, MA, USA) were cultivated in commercial Expi293 expression medium (Thermo Fisher Scientific, Waltham, MA, USA) at 37 °C, 8% CO_2_, and 90 rpm agitation. HEK-293T-ACE2 cells (a kind gift from Prof. Stephan Pöhlmann, Göttingen, Germany) were cultivated in DMEM (Gibco/Thermo Fisher Scientific, Waltham, MA, USA) supplemented with 10% fetal bovine serum and 1% Penicillin/Streptomycin (both from Pan Biotech, Aidenbach, Germany). Every fourth passage, the medium was supplemented with 1 μg/mL Puromycin (InvivoGen, San Diego, CA, USA). HEK-293T-ACE2 cells were cultivated at 37 °C and 5% CO_2_.

### Recombinant Proteins

The receptor-binding domain (RBD, residues 319–532) of SARS-CoV-2 Spike proteins was expressed and purified as previously described.^25^ Briefly, the coding sequences, together with an N-terminal minimal tPA signal peptide and a C-terminal Avi-hexahistidine tag, were cloned into a pcDNA5/FRT/TO-derived expression plasmid. Additionally, an RBD Alpha containing an N-terminal CAAC tag and a flexible (G_4_S)_3_ linker (NtCC)^26^ between the signal peptide and the RBD sequence was generated, similar to NtCC-RBD.^27^ Expression of recombinant proteins was performed using the commercial ExpiFectamine 293 transfection kit (Thermo Fisher Scientific, Waltham, MA, USA). Soluble RBDs were purified 5 days post-transfection via immobilized-metal affinity chromatography using HisTrap Excel columns (Cytiva, Marlborough, MA, USA) on an FPLC device (Äkta, Cytiva, Marlborough, MA, USA) with a linear gradient of 10–500 mM imidazole in phosphate-buffered saline (PBS; Cat. No. 14190-094, Thermo Fisher Scientific, Waltham, MA, USA) gradient. RBD of Omicron variants BA.2, BA.5, and BQ1.1 were additionally polished with a size exclusion chromatography step. Four mg of Omicron RBD at 4 mg/mL were loaded onto a Superdex 75 Increase 10/300 GL column (Cytiva, Marlborough, MA, USA), operated on an FPLC device (Äkta, Cytiva, Marlborough, MA, USA) in PBS at a flow rate of 0.8 mL/min. RBD-containing fractions were detected using reducing SDS-PAGE. The calculated molecular weight (MW) and isoelectric points (pI) can be found in Table S1.

Human ACE2 was expressed and purified as described previously.^28^

### SDS-Page

For SDS-PAGE analysis the indicated amount of protein was heated at 95 °C for 10 min in 1× reducing SDS-PAGE buffer and loaded onto a self-cast 12.5% polyacrylamide gel (https://pubmed.ncbi.nlm.nih.gov/5432063/). The gel was run for 80 min at 140 V. Staining was done with Coomassie staining solution (50% (v/v) ethanol, 10% (v/v) acetic acid, 0.25% (w/v) Coomassie Brilliant Blue G250 in H_2_O) for 20 min and destained in 7% (v/v) acetic acid solution.

### Biotinylation of RBD

Proteins were biotinylated with different approaches: either enzymatically with BirA, chemically in an undirected manner targeting amines, or chemically in a site-directed manner targeting the N-terminal Cys-tag (NtCC).

RBD proteins were site-specifically biotinylated at the C-terminal Avi-hexahistidine tag using the BirA biotin-protein ligase kit (Avidity), as described previously.^29^ Thirty nmol of protein, at a final concentration of 100 μM in PBS, supplemented with 10 mM ATP, 10 mM Mg­(OAc)_2_, and 150 μM dbiotin, were biotinylated for 2 h at 30 °C with 7.5 μg BirA. Biotinylated proteins were buffer exchanged into PBS via centrifugation in Amicon ultrafiltration devices three times to ensure the separation of free biotin and ATP. Efficiency of biotinylation was monitored using a neutravidin shift assay. Briefly, 50 pmol of enzymatically biotinylated protein was denatured at 95 °C for 10 min in a reducing SDS-PAGE buffer. After cooling to RT, either 150 pmol of neutravidin or the same volume of PBS was added and subjected to SDS-PAGE analysis.

NHS-biotin was dissolved in DMSO (5 mg/mL) and diluted 1:10 in PBS (0.5 mg/mL). RBD was mixed with different equivalents of NHS-biotin and incubated for 2 h at 22 °C and 300 rpm in a Protein LoBind cup (Eppendorf, Hamburg, Germany). Samples were diluted with PBS (total volume ∼400 μL) and purified via centrifugal filtration in 10 kDa Pierce concentrators (Thermo Fisher Scientific, Waltham, MA, USA) at 12000 RCF for 5 min with 4 washing steps (400 μL PBS). Finally, the biotinylated proteins were recovered and stored in Protein LoBind tubes. Concentrations were determined using a NanoDrop One (Thermo Fisher Scientific, Waltham, MA, USA) (*M*_w_(RBD) = 26.97 kDa, ε­(RBD) = 40.34).

Site-specific biotinylation was undertaken at the Cys residues of the protein N-terminus. A short (PEG2) and long (3 kDa/PEG-68) PEG linker was chosen. The short linker was prepared by dissolving 5.3 mg (0.014 mmol) of biotin-PEG_2_-amine in 1 mL of dry and degassed DMF; then 3.25 mg of Mal-NHS (0.012 mmol) were added, vortexed, and reacted for 1 h at room temperature in the dark. The product (Mal-PEG_2_-Biotin) was used without further purification as a frozen stock in a DMF solution.

Long and short linkers were both coupled under identical conditions. To 350 μL of RBD in PBS solution, 125 μg of TCEP was added to achieve a final concentration of 2.5 mM and reacted for 30 min. Afterward, a 50-fold excess of Maleimide-Biotin-PEG and Mal-PEG_2_-Biotin, respectively, was added and incubated for 16 h at room temperature. Biotinylated protein was purified and concentrated by buffer exchange using Amicon 10 kDa centrifugal filters. Characterization was carried out via the HABA-Avidin Assay for biotin content using a colorimetric biotin assay kit (MAK171, Sigma-Aldrich/Merck, Darmstadt, Germany). Protein concentration was determined via UV absorbance.

### Antisera

SARS-CoV-2-positive sera were obtained by using samples from the prospective longitudinal multicenter cohort study (CoVaKo) in which acute SARS-CoV-2 BTIs and non-BTIs were analyzed. The study centers were the University Hospitals in Erlangen, Regensburg, Augsburg, Würzburg, and Munich (TUM and LMU), all located in Bavaria, Germany. The study design and cohort composition have been thoroughly described in Prelog et al.^30^ and Einhauser et al.^31^ Additionally, seropositive samples were obtained from the TiCoKo19 cohort, previously described in Wagner et al.^32^ and Einhauser et al.^33^

In particular, sera were selected to provide a variety of immune profiles and immunization backgrounds that resemble a real-world testing scenario. Thus, sera were selected for (wild-type) vaccination, sometimes combined with Delta breakthrough infection or vaccination (wild-type and Omicron-adjusted) and Omicron breakthrough infection.

The TiKoCo study was approved by the Ethics Committee of the University of Regensburg, Germany (vote 20-1867-101) and adopted by the Ethics Committee of the University of Erlangen (vote 248_20 Bc). The CoVaKo study was approved by the Ethics Committee of the Friedrich-Alexander-University Erlangen-Nürnberg, Germany (vote 46_21 B) and adopted by the local ethics committees of all other study centers. The CoVaKo Clinical Trials registration number was DRKS00024739. All study participants provided written informed consent. Both studies, TiCoKo19 and CoVaKo19, comply with the 1964 Declaration of Helsinki and its later amendments.

Seronegative prepandemic anonymized plasma samples from healthy adult blood donors (‘BRK···’) were purchased from the Bavarian Red Cross. Pooled Human Complement Serum (IR45270 and IR46827) was obtained from Innovative Research (Novi, MI, USA).

### Liposome Preparation

Reverse-phase evaporation was chosen as the preparation method for liposomes, as described previously.^34^ 150 mM concentration of SRB and 140 mM NaCl were dissolved in 20 mM HEPES, pH 7.5 (4.5 mL), by sonication at 60 °C to prepare the encapsulant. Lipid mixtures (60 μmol containing 41.4 mol % cholesterol, 32.2 mol % DPPC, 18.4 mol % DPPG, and 8.0 mol % *N*-glutaryl-DPPE) were prepared by the addition of 3 mL of chloroform and 0.5 mL of methanol and sonication for 1 min, followed by the addition of encapsulant (2 mL) and sonication for 4 min at 60 °C. A rotary evaporator (LABOROTA 4001, Heidolph, Germany) was used to evaporate the organic solvents at 60 °C by stepwise reduction of pressure (900 mbar for 10 min, 850 mbar for 5 min, 800 mbar for 5 min, and 780 mbar for 20 min). The solution was vortexed another two times for 1 min with intermittent encapsulant addition (2 mL). The residual organic solvents were evaporated at 60 °C (750 mbar for 20 min, 600 mbar for 5 min, 500 mbar for 5 min, and 400 mbar for 20 min). The size was controlled by extrusion at 65 °C using polycarbonate membranes with pore sizes of 1, 0.4, and 0.2 μm. Solutions were repeatedly pushed through the membranes with decreasing pore sizes, amounting to 21 repetitions for the 1 μm pore size and 11 repetitions for each of the smaller pore sizes. Size exclusion chromatography using a Sephadex G-50 column, followed by dialysis overnight against HSS buffer with 2 buffer exchanges in a dialysis membrane Spectra/Por© 4 (MWCO: 12–14 kDa), was used to remove excess encapsulant.

### Characterization of Liposomes

An inductively coupled plasma optical emission spectrometer (ICP-OES) (SpectroBlue TI/EOP) from SPECTRO Analytical Instruments GmbH (Kleve, Germany) was used to determine phospholipid concentrations, which, in turn, were used to calculate total lipid concentrations based on the mixture of lipids used for the preparation. Phosphorus standard dilutions between 0 and 100 μM in 0.5 M HNO_3_ were used for calibration of the device. Phosphorus was detected at 177.495 nm. Recalibration was performed before each measurement using the 0 and 100 μM phosphorus dilutions. Liposome stock solutions were diluted 1:100 or 1:60 in 0.5 M HNO_3_ and their phosphorus content determined.

Size and ζ-potential were measured via dynamic light scattering (DLS) using a Malvern Zetasizer Nano-ZS. Liposome stock solutions were diluted to 25 μM total lipids in HSS buffer in a poly­(methyl methacrylate) (PMMA) semimicro cuvette (Brand, Germany) for size measurements and a disposable folded capillary cell (Malvern Panalytical, Germany) for ζ-potential measurements. The measurement temperature was set to 25 °C, the refractive index was 1.34, the material absorbance was zero, and the dispersant viscosity was 1.1185 mPa s. For ζ-potential measurements, a dielectric constant of 78.5 was used, and an equilibration time of 60 s was applied before each measurement.

### Modification of Liposomes

Proteins were conjugated to carboxylated liposomes via EDC/sulfo-NHS chemistry. Liposomes were incubated with EDC and sulfo-NHS (1:100:180 ratio of carboxy-groups:EDC:sulfo-NHS) for 1 h at room temperature (RT) and 300 rpm, followed by the addition of protein and another 1.5-h incubation at RT and 300 rpm. Excess reagents were removed via dialysis against HSS buffer overnight with one buffer exchange in a Spectra-Por Float-A-Lyzer G2 (1 mL, MWCO: 1000 kDa) for large volumes or via size exclusion chromatography with Sephadex G-50 or G-100 for small volumes (<50 μL). Total lipid concentrations were determined using ICP-OES, and the conjugated liposomes were stored at 4 °C in Protein LoBind tubes (Eppendorf, Germany).

### Heterogeneous Binding Assays

Proteins (1 μg/mL ACE2 or 2 μg/mL antibodies in PBS, 100 μL per well) were incubated in a high-binding microplate overnight at 4 °C. The plate was emptied and blocked for 1 h at 300 rpm with 1 w/v% BSA in PBS-T (150 μL per well). It was washed two times with PBS-T (150 μL/well) and three times with HSS (150 μL/well) before being used. Liposomes (1 μM total lipids unless stated otherwise) (mixed with RBD-biotin in the case of streptavidin/neutravidin-liposomes) were added to the microtiter plate (MTP) (100 μL per well, *n* = 3) and incubated for 2 h at RT and 300 rpm. The plate was washed three times with HSS buffer (150 μL per well) before 30 mM OG in bidest. water (100 μL per well) was added. After 10 min of incubation, the fluorescence was measured three consecutive times with a BioTek SYNERGY Neo2 fluorescence reader (Agilent Technologies, Santa Clara, CA, USA) (λ_Ex_ = 560 nm and λ_Em_ = 585 nm, bandwidth 10, gain 150).

### Measurement of Unlysed Fluorescence

Liposomes were diluted in HSS (5 μM total lipids) and added to a black 96-well microplate (100 μL per well, *n* = 4). The fluorescence was measured three consecutive times with a BioTek SYNERGY Neo2 fluorescence reader (λ_Ex_ = 560 nm and λ_Em_ = 585 nm, bandwidth 10, gain 100). Liposomes were lysed by the addition of 300 mM OG (10 μL per well) and incubated for 10 min at RT and 300 rpm before the fluorescence was measured again. Unlysed fluorescence was calculated by normalizing the fluorescence intensity of the liposomes before the addition of OG (unlysed) to that after incubation with OG (lysed). Errors were calculated by using Gaussian error propagation. The encapsulation efficiency was determined from the lysed fluorescence and a calibration curve of SRB in 30 mM OG, as described previously.^35^

### Surrogate Virus Neutralization Test

ACE2-biotin (1 μg/mL in PBS, 100 μL per well) was incubated in a streptavidin microplate for 1 h at RT and 300 rpm. The plate was emptied and blocked for 1 h at 300 rpm with 10 μM biotin in PBS-T (150 μL per well). It was washed two times with PBS-T (150 μL per well) and three times with HSS (150 μL per well) before use. Streptavidin/neutravidin-liposomes (1 μM total lipids unless stated otherwise) were mixed with RBD-biotin (25 ng/mL unless stated otherwise) and serum (0 to 4 v%) and incubated for 1 h at 30 °C and 300 rpm. Samples were added to the MTP (100 μL per well, *n* = 3) and incubated for 2 h at RT and 300 rpm. The plate was washed three times with HSS buffer (150 μL per well) before 30 mM OG in bidest. water (100 μL per well) was added. After 10 min of incubation, fluorescence was measured three consecutive times with a BioTek SYNERGY Neo2 fluorescence reader (λ_Ex_ = 560 nm and λ_Em_ = 585 nm, bandwidth 10, gain 150). Binding inhibition, expressed as a percentage, was calculated as (1 – fluor. int./fluor. int. lowest serum dilution) × 100. IC50 values were obtained via logistic fit with 0% as the lower limit and 100% as the upper limit, with no weighting performed, using the Origin 2020 software.

### Pseudovirus Neutralization Test

Pseudovirus neutralization for SARS-CoV-2 was performed as described previously.^36,37^ In brief, an inoculum containing 2.5 × 10^5^ RLU/384-well of lentiviral SARS-CoV-2-Spike pseudotypes expressing luciferase was neutralized using a 2-fold serum dilution series starting at 1:20 for 1 h. After 48 h of infection of HEK293T-ACE2-positive cells, luciferase activity was determined using the Bright-Glo reagent (Promega Corp., Madison, WI, USA). The 50% inhibitory dilution (ID50) of the sera was calculated in GraphPad Prism 8 (San Diego, CA, USA) by normalizing the data to both infected and noninfected cells, followed by curve fitting with the “log (inhibitor) vs. normalized response” algorithm. Neutralizing antibody titers were assessed against various SARS-CoV-2 variants, namely Alpha (B.1.1.7), Delta (B.1.617.2), and Omicron BA.2, BA.5, and BQ.1.1.

### Antigenic Landscaping

Due to the limited number of sera measured in this manuscript, the antigenic map footprint was taken from visit four of Einhauser et al.^31^ As described there, the antigenic maps were computed using the Racmacs package with 1000 optimizations. Antigens not measured within this article were removed from the maps. Antibody landscapes were generated by adding a third dimension to the antigenic map represented by the geometric mean titers to each variant and subsequently fitting a generalized additive model (GAM)^38^ to the variant-specific titer values and the corresponding antigenic coordinates. This was used as a more flexible alternative to the traditional ablandscapes package’s cone-shaped object calculation, as no WT titers were available for the cone peak. GAMs were computed using the mgcv package. Landscapes were visualized in 3D using the r3js package.^39^

### Data Evaluation and Statistical Analysis

Data were analyzed statistically by using OriginPro 2024 software. To compare three or more different samples, a one-way analysis of variance (ANOVA) with a post hoc Tukey’s test was performed. *p*-values ≤0.05 were considered statistically significant. *p ≤ 0.05, **p ≤ 0.01, ***p ≤ 0.001, and ns = not significant.

## Results and Discussion

### Conventional EDC/Sulfo-NHS Coupling of RBD to COOH-Liposomes

Modification of fluorescent, 150 mM SRB-encapsulating, COOH-liposomes with RBD using EDC and sulfo-NHS chemistry worked well for Alpha, as shown previously,^28^ and Delta, but not for Omicron variants. In the ACE2-binding assay, the RBD-modified liposomes are allowed to bind to ACE2 immobilized in the MTP. Nonbound liposomes are washed away, and finally, the fluorescence of the SRB released upon lysis of bound liposomes with OG is measured. Among the Omicron variants, RBD-BA.2- and RBD-BQ1.1-liposomes showed lower binding, and RBD-BA.5-liposomes showed no binding to the recombinant ACE2 receptor (Figure 1A). Additionally, the successful coupling of all variants except for RBD-BA.5 was proven by both increased hydrodynamic diameter and surface charge of liposomes, as demonstrated by DLS and ζ-potential measurements (Figure S1 and Table S2). Identical mole fractions of each RBD variant were added during coupling (0.2 mol %), but the amount of RBD actually coupled to the liposomal surface was not determined and might vary for each variant. Interestingly, although RBD-BA.5 showed neither differences in size or ζ-potential following EDC/sulfo-NHS coupling to liposomes nor binding to ACE2, RBD-BA.5-liposomes still bound to immobilized polyclonal anti-RBD antibodies targeting multiple epitopes on the RBD^40^ (Figure 1B). This suggests an unfavorable orientation of RBD-BA.5 on the liposomal surface, preventing its proper interaction with ACE2. In conclusion, it could be assumed that the coupling reaction is less efficient due to the absence of proof of coupling via DLS measurements and the low signals obtained upon binding by the immobilized antibodies.

**Figure 1:**
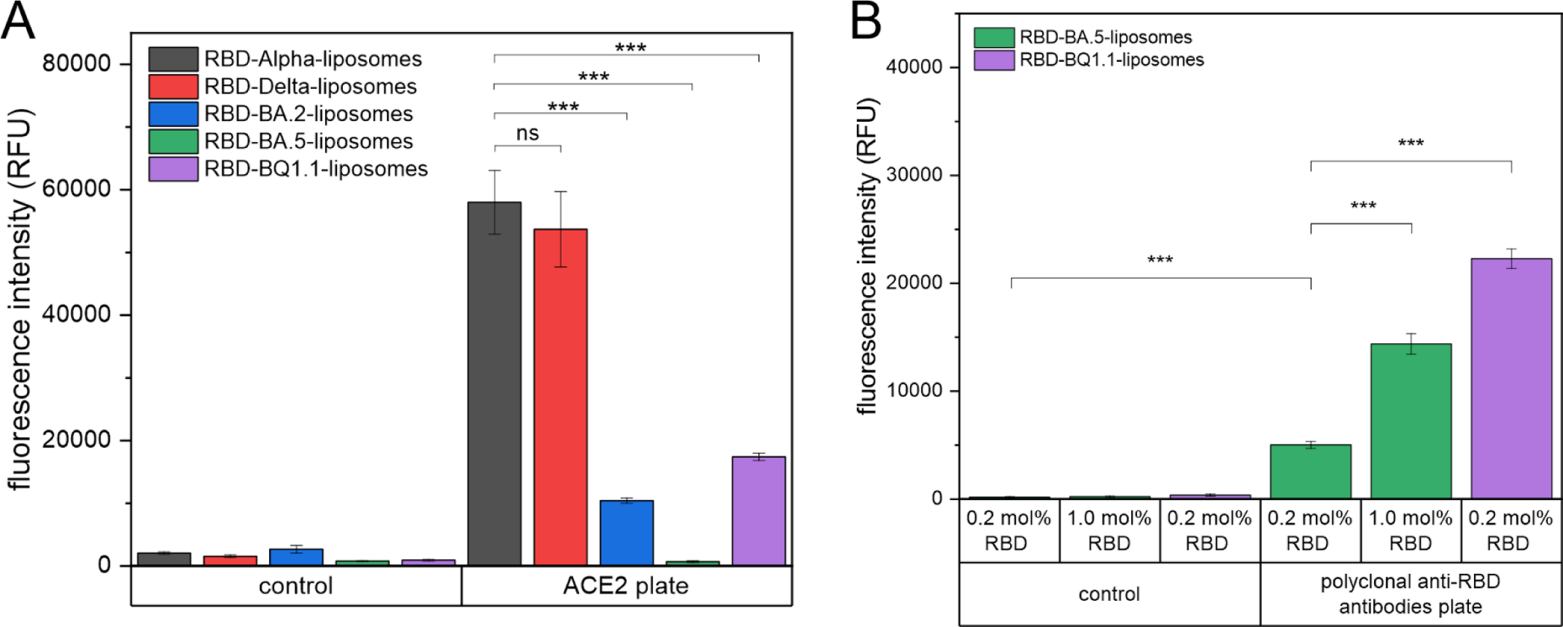
(A) ACE2 binding of liposomes modified with 0.2 mol % of different RBD variants. *n* = 3 (^***^ *p* < 0.001, ns = not significant). (B) Polyclonal anti-RBD antibody binding of liposomes modified with 0.2 or 1.0 mol % of RBD-BQ1.1 and BA.5 variants. *n* = 3 (^***^ *p* < 0.001).

To substantiate the hypothesis of a less efficient coupling reaction of RBD-BA.5 to liposomes, we compared the signals of RBD-BA.5- and RBD-BQ1.1-liposomes that were fabricated with varying amounts of the respective RBD (Figure 1B). Here, 1.0 mol % RBD-BA.5 generated even lower signals than 0.2 mol % RBD-BQ1.1 (one-way ANOVA, *p* < 0.001). The altered physicochemical properties, characterized by a higher electropositive surface of Omicron RBD variants,^24^ may result in changed interactions with other surfaces, like liposomes, particularly due to nonuniform distribution of the lysine residues on the RBD surface, which is further aggravated by the use of a nondirected coupling strategy.

Therefore, we hypothesize that the location of the respective amino acids accessible for the coupling reaction strongly influences the coupling efficiency. Specifically, EDC and sulfo-NHS react with COOH-groups on the liposomes, producing an amine-reactive sulfo-NHS-ester that subsequently reacts with NH_2_-groups of the protein, typically targeting the N-terminal α-amino group and the ε-amino group of lysines.^14^ Alpha-RBD has 10 lysine residues (see Figure S2), of which three are located in (marked gray) or next to (marked with red arrows) the ACE2-binding motif. Since coupling via EDC/sulfo-NHS chemistry works well, it can be safely assumed that these lysine residues are unlikely to react with the sulfo-NHS-ester. Instead, the other lysines are favored, resulting in the proper orientation of the protein and enabling binding to ACE2. Aligning the sequences of the five RBD variants reveals an abundance of mutations, especially between the Alpha/Delta and the Omicron variants (Table S3). Delta differs from BA.2 in 16 positions, including the substitution of a lysine residue (K417N) and the introduction of a new lysine residue (N440 K). A possible explanation for the decreased ACE2 binding by RBD-BA.2-liposomes might be the unfavorable orientation of RBD-BA.2 during coupling compared to Alpha and Delta.

Only 3 mutations differentiate RBD-BA.2 and -BA.5, none of which replace or introduce a lysine they do introduce an arginine (L452R), which might alter the shielding of an already present lysine, causing it to become the preferred lysine for EDC/sulfo-NHS coupling, resulting in an unfavorable orientation of RBD-BA.5, and hence completely preventing its interaction with ACE2. For RBD-BQ1.1 a lysine residue adjacent to the ACE2-binding motif is replaced (K444T), while a new one is introduced outside of it (N460K). If the latter is favored for the reaction with the sulfo-NHS-ester, it could explain why RBD-BQ1.1-liposomes are able to bind to ACE2 again, whereas RBD-BA.5-liposomes are not. Even though the difference in lysine residues among all RBD variants is minimal, the nucleophilic properties of lysine are strongly influenced by the surrounding microenvironment.^41^ Therefore, changes in the proximity of lysines can alter their reactivity with sulfo-NHS-esters. A similar effect is observed with the α-amino group in direct proximity to multiple histidines, where the high density of imidazole side chains foster the reaction with 4-methoxyphenyl-esters under physiological conditions.^42^ Another possible mechanism that could contribute to the different coupling orientations, might involve the location of arginines (Q493R), which could complex with the sulfo-NHS-ester and preferentially foster reactions with adjacent lysines rather than with the α-amino group of the N-terminus. Furthermore, arginine might influence the orientation of RBD by electrostatic interactions with the negatively charged lipid bilayer, increasing the likelihood of surrounding lysines reacting with the sulfo-NHS-ester.

The reaction of sulfo-NHS-esters with α-amino groups is favored around pH 7, while pH 8 enhances reactivity toward ε-amino groups of lysines.^15^ When pH 8 was used during the coupling reaction, surprisingly, decreased ACE2-binding ability for all variants was observed (data not shown). This suggests that the higher pH made some of the lysine residues located within or adjacent to the receptor-binding motif more reactive, rather than those outside of it, bolstering our hypothesis.

There are conflicting data in the literature regarding the affinity of ACE2 with the different VOCs, mainly influenced by different methods, either equilibrium-based or kinetic-based measurements, or variations in the assay setup, and the use of either monomeric RBD or trimeric prefusion-stabilized Spike protein.^17,43–47^ All in all, the binding of ACE2 to the different VOCs seems to be in a similar range. However, this was not reflected by the RBD-liposomes decorated with RBD using EDC/sulfo-NHS chemistry, where RBD-Alpha- and RBD-Delta-liposomes performed significantly better compared to RBD-BA.2- and RBD-BQ1.1-liposomes (one-way ANOVA, *p* < 0.001). This discrepancy is assumed to be due to different orientations on the liposomes, which resulted from the coupling chemistry and mutations within the variants. We conclude that sequence variations strongly impact the coupling efficiency and orientation of proteins using EDC/sulfo-NHS chemistry, and predictions based on the type and location of the mutations are very difficult. It is therefore advisable to focus on directional coupling strategies that do not rely on the use of amino acids present throughout the protein peptide chain.

Protein production through recombinant and cell culture technologies relies on the cells’ ability to produce a stable protein, as well as on purification and storage processes to maintain the stability of protein solutions. Aggregation is a frequently observed phenomenon caused by intrinsic factors, e.g., the protein structure and extrinsic factors, e.g., the environment during expression, purification, and storage.^48^ Here, the investigation of the thermal stability of RBD variants by NanoDSF revealed that each RBD variant exists in a different state of multimerization (Figure S3A) and that RBD-BA.2 and RBD-BA.5 have lower melting temperatures compared to RBD-Alpha, RBD-Delta, and RBD-BQ1.1 (Figure S3B). It remains unclear whether this affects coupling or if the multimers disband into monomers during coupling due to the lower concentration, different environment (e.g., buffer), and shaking. This phenomenon will be further investigated in the future. However, stability during storage was examined using RBD-Alpha-modified liposomes (Figure S4A). Storage stability for up to 24 weeks was achieved when stored in PBS with an additional 200 mM sucrose to ensure liposome stability and 0.04 w/v % HSA for RBD stability. Furthermore, it was found that storage at higher concentrations (100 μM) vs lower concentrations (25 μM) enhanced RBD-liposome stability, as the latter showed a significant signal loss in the ACE2-binding assay after just 12 weeks, whereas the former showed signal loss only after 36 weeks (one-way ANOVA, *p* < 0.001).

### Alternative Biotinylation Strategies for Random or Site-Directed Coupling of RBD to Streptavidin-Liposomes

In prior research, liposomes modified with streptavidin (stav-liposomes) had been optimized and demonstrated to be stable for years at 4 °C.^10^ Furthermore, random and site-directed biotinylation of proteins are well established.^49,50^ These strategies and their effects on RBD variants were investigated here to find a more general alternative to replace EDC/sulfo-NHS-assisted protein coupling. More precisely, biotinylation of NH_2_-residues using NHS-biotin, directed biotinylation of an N-terminal Cys-tag, and enzymatic biotinylation of the C-terminal Avi-tag were compared to EDC/sulfo-NHS strategy optimized for the Alpha variant, which initially worked sufficient.^28^ A critical issue to overcome in such an approach is the additional binding event between streptavidin and biotin, affected by the location and accessibility of the biotin tag, which can easily lead to a lower surface coverage than the direct immobilization approach.

In line with previous observations, the reaction of NH_2_-groups with NHS-esters, in this case NHS-biotin, is generally feasible, as shown using RBD-Alpha, RBD-BA.5, and RBD-BQ1.1 (Figure S5). Interestingly, this approach even enabled the binding of RBD-BA.5-biotin-modified stav-liposomes to ACE2, which was not feasible with standard EDC coupling as shown above, which even bolstered the suggested unfavorable *ab initio* interaction with the liposome surface, leading to an incorrect orientation of RBD-BA.5 on liposomes. Random biotinylation of amino groups, instead of direct coupling to the liposome, slightly improves the orientation of RBD-BA.5, thereby enabling interaction with ACE2. However, the use of 100 equiv of NHS-biotin resulted in excessive biotinylation of all three variants, preventing interaction with ACE2 for all of them. Therefore, the ratio between RBD and NHS-biotin was further optimized for the Alpha variant, where ratios of 1:2 and 1:5 of RBD to NHS-biotin appeared to work better than 1:1 and 1:10 ratios (Figure S6). The highest signals were obtained with 75 ng/mL RBD-biotin (5 equiv), followed by a hook effect at higher concentrations, and 100 ng/mL RBD-biotin (2 equiv), which reached a plateau. While a 1:1 ratio is insufficient to biotinylate each RBD molecule, higher ratios appear to result in multiple, and likely inhomogeneous, biotinylations of each RBD, increasing the likelihood of blocking lysine residues that contribute to the ACE2-binding interface. When compared to site-directed biotinylation strategies, the NHS-biotin approach produced by far the lowest signal intensities (Figure 2A).

**Figure 2:**
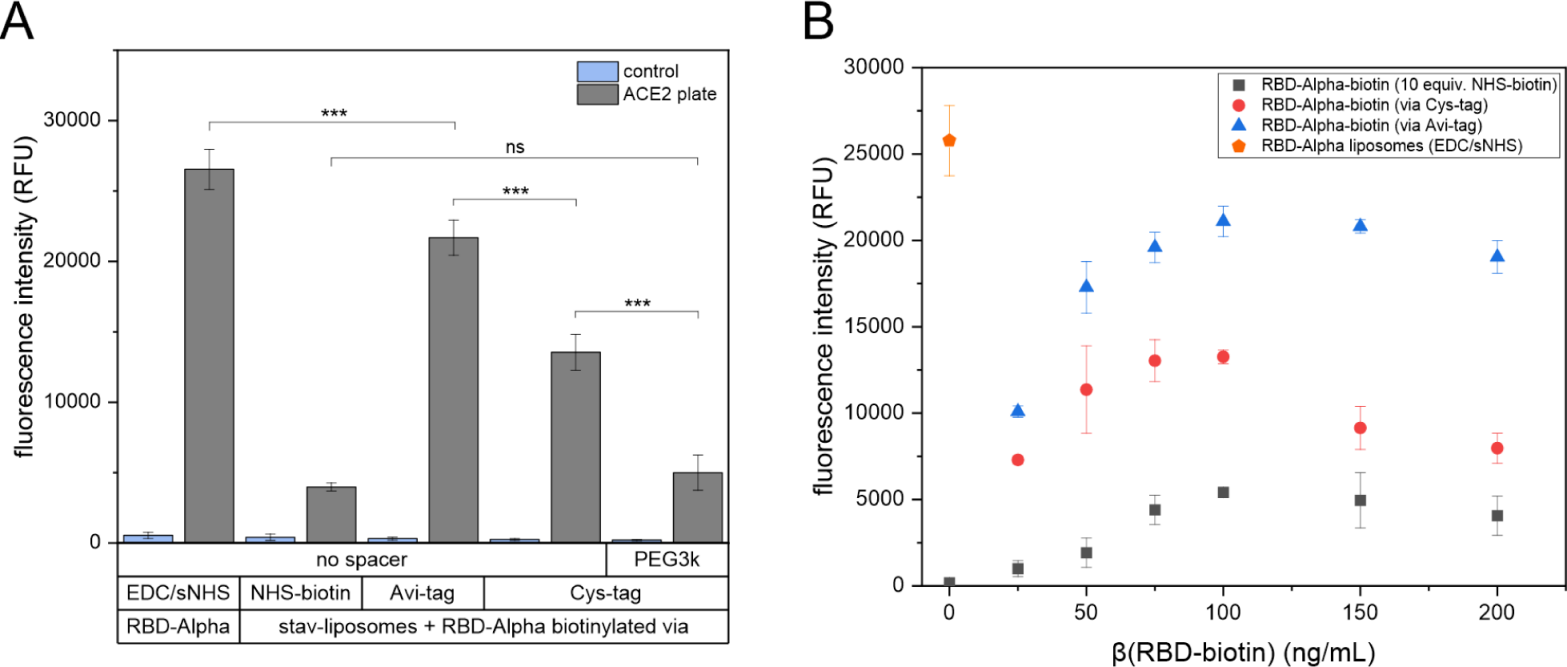
(A) ACE2 binding of RBD-Alpha-liposomes (EDC/sNHS) and streptavidin-liposomes with various RBD-Alpha-biotin conjugates. *n* = 3 (^***^ *p* < 0.001, ns = not significant). (B) ACE2 binding of streptavidin-liposomes plus RBD-Alpha-biotin modified using 10 equiv. NHS-biotin (10×) or biotinylated via the Cys-tag or the Avi-tag. *n* = 3.

The modifications via Avi- or Cys-tag enable single, homogeneous, and site-directed biotinylation of RBD molecules. While this limits each RBD to just one biotin moiety and hence, in theory results in lower efficiency in binding to streptavidin, both tags have the clear advantages of not involving any critical lysine residues within the RBD molecule. Additionally, they display the RBD molecule with perfect orientation on the liposome surface and generate higher reproducibility for future applications in diagnostic assays. For coupling via the Cys-tag, various commercially available biotin-maleimide conjugates can be used, including those with spacers. Spacer-free conjugates and those including a 3-kDa polyethylene glycol (PEG) spacer were tested. The latter contains 68 monomers and has a theoretical length in water of 19 nm.^51^ Interestingly, liposomes tagged with RBD that was biotinylated spacer-free at the Cys-tag showed more than double the binding efficiency to ACE2 compared to those using the PEG spacer. This can, in part, be explained by the lower degree of biotinylation achieved with the PEG spacer (0.6 ± 0.2 biotin per RBD) compared to no spacer (1.5 ± 0.5 biotin per RBD), as confirmed by a HABA assay. Degrees above one biotin per RBD are possible due to the two individual cysteines constituting the Cys-tag, but they are not thought to be beneficial. Besides the lower degree of biotinylation, the long spacer might sterically interfere with the ACE2 interaction. For other proteins, the introduction of a spacer might be favorable, though this would need to be specifically tested.

The Avi-tag consists of 15 amino acids that are recognized by *E. coli* biotin ligase (BirA), which biotinylates a specific lysine residue within the recognition site.^52^ The advantages of this approach are the natural spacer afforded by the remaining amino acids and the high consistency of the enzymatic reaction. An additional benefit is the potential combination with a His-tag, typically used for protein purification using Ni-NTA affinity chromatography,^53^ necessitating only a single engineering step of the corresponding DNA sequence. Successful enzymatic biotinylation was verified with an SDS-PAGE-based neutravidin shift assay, indicating quantitative biotinylation for Delta, BA.2, and BA.5, and almost quantitative biotinylation of Alpha and BQ1.1 RBD variants (Figure S7). Finally, by varying the concentration of biotinylated RBD used for binding to the liposomes, it was shown that in all scenarios, an optimal RBD concentration can be found at 100 ng/mL, but only the modification with the Avi-tag allows signals of the same strength as the direct EDC/NHS coupling of the Alpha RBD variant (Figure 2B). It must be assumed that only the C-terminal afforded orientation on the liposomes leads to favorable interactions with ACE2, re-enacting the orientation of RBD within the context of Spike on the virus membrane of SARS-CoV-2. Therefore, this strategy was applied to all RBD variants, and successful binding to ACE2 immobilized on a microtiter plate was demonstrated, even for RBD-BA.5 (Figure S8). Finally, and rather surprisingly, not all variants behaved the same with the blocking reagents used, as BA.2 demonstrated strong nonspecific binding to BSA. In the end, skim milk powder was identified as a blocking agent that can be used equally well for all variants, avoiding nonspecific binding (Figure S9).

### Establishing the Surrogate Virus Neutralization Test

For the development of a surrogate virus neutralization test, a competitive assay format was developed in which antibodies inhibit RBD-liposomes from binding to ACE2 immobilized on a microtiter plate. Neutralizing antibodies present in a sample would interfere with this binding and lead to a lower signal. The assay principle has proven to be a valid surrogate in an ELISA format^54^ and had already been successfully established for the Alpha variant.^28^ Initial studies demonstrated the importance of ACE2 orientation for immobilization, suggesting that only in the oriented format using biotinylated ACE2 are sufficient binding sites available to promote efficient binding between RBD-liposomes and ACE2 in this heterogeneous format. This effect was identified as the presence of prepandemic serum, which would disturb the binding of all types of RBD-liposomes to randomly immobilized ACE2, but not to ACE2-biotin immobilized site-directed (Figure S10 shows exemplary data for RBD-Alpha-liposomes). Blocking of streptavidin plates with biotin after immobilization of ACE2-biotin enabled their use for the new RBD-biotin approach, improving stav-liposome capture.

Second, liposomes were further optimized to serve as a truly general reagent for the immobilization of biotinylated proteins. Since streptavidin contains the amino acid sequence RYD (Arg-Tyr-Asp), which is known to interact with the sequence RGD (Arg-Gly-Asp) present in proteins,^55^ neutravidin-liposomes (nav-liposomes) were investigated as an alternative. Neutravidin is a deglycosylated version of avidin and does not contain the RYD sequence. Interestingly, even though both ACE2 and RBD contain the RGD sequence, neither stav- nor nav-liposomes were captured on an ACE2-biotin-coated plate in the presence of free RBD (50 to 200 ng/mL) (Figure S11). This suggests that the sequences are hidden within the protein structures (D in RGD motif in ACE2 is facing the inner core of protein) or the binding kinetics are simply unfavorable, which is probably the case for RGD in RBD Alpha and Delta, where the RGD motif is exposed on the surface, according to the crystal structure 6vw1 in the PDB repository. Both stav- and nav-coated liposomes reacted similarly to seronegative and seropositive samples (Figure 3A,B), demonstrating the applicability of both liposome types. To obtain a universally applicable platform technology and to prevent serum reactivity against streptavidin,^56^ nav-liposomes were used for all subsequent studies. These also showed a slightly higher IC50 (84 vs 69) compared to the stav-liposomes (Figure 3B), which implies higher sensitivity, as identical amounts of neutralizing antibodies cause slightly stronger neutralization.

**Figure 3:**
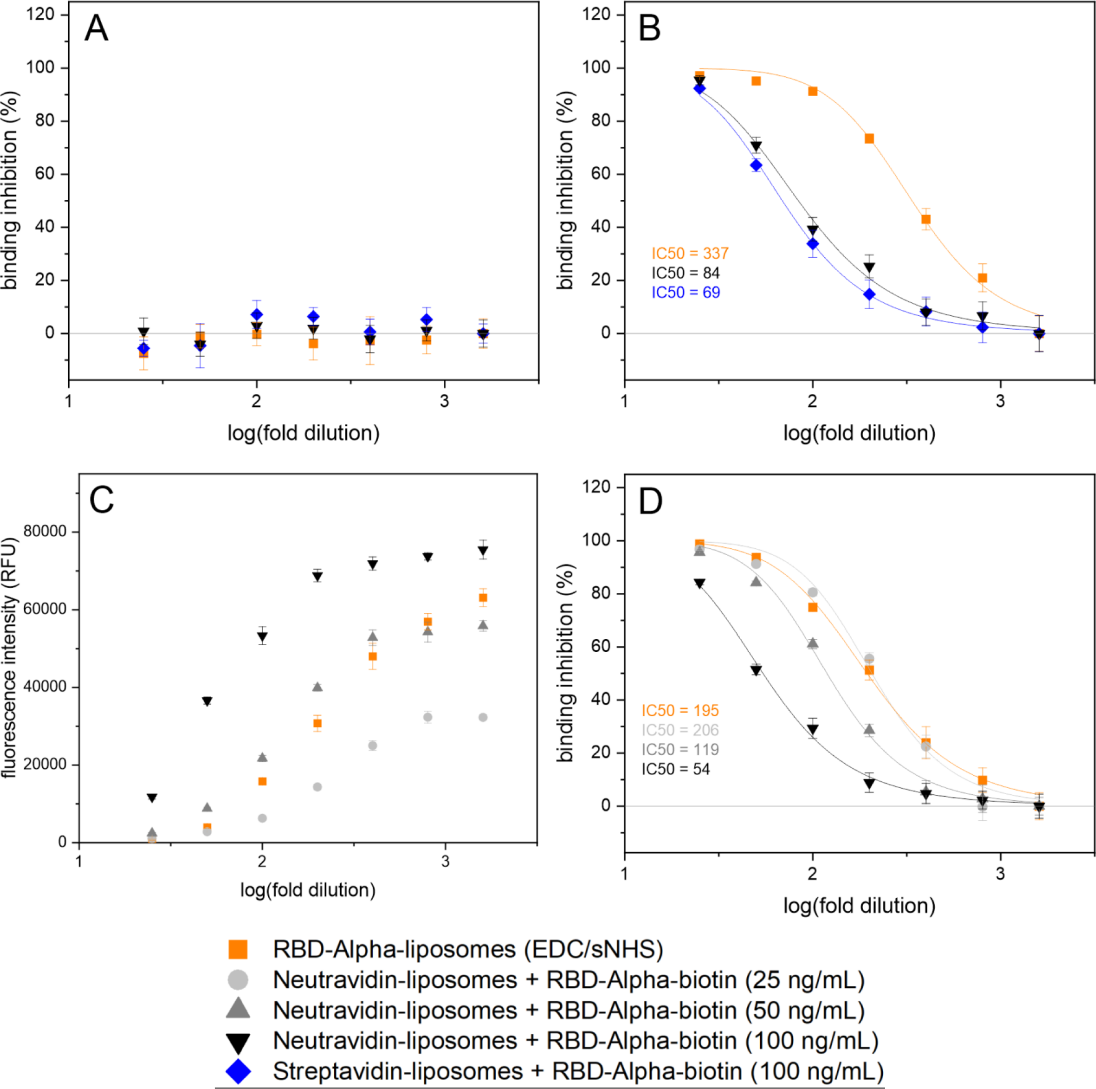
Neutralization tests of RBD-Alpha-liposomes and streptavidin- or neutravidin-liposomes plus RBD-Alpha biotinylated via the Avi-tag, with a prepandemic serum (BRK4500) (A), seropositive sample IR46827 (B), or seropositive sample IR45270 (C and D). RBD-Alpha-liposomes were used at a concentration of 1 μM for (B) and 0.5 μM for (C and D). *n* = 3.

RBD-Alpha and pooled seropositive samples were used for fine-tuning assay conditions. In a competitive assay format, the concentrations of the binding partners play a crucial role in the obtainable limits of detection and need to be balanced with the signals obtained via the liposome concentration itself. The comparison of RBD-liposomes, RBD-nav-liposomes, and RBD-stav-liposomes with the seropositive sample (IR46827) had revealed lower IC50 values for the format with stav/nav-liposome plus RBD-Alpha-biotin (69 and 84, respectively) compared to just RBD-Alpha-liposomes (337) (Figure 3B). This can be explained by the approximately two times higher RBD loading for the stav/nav-liposome format, while the input liposome concentration was kept constant for the assay. Both formats produced identical IC50 values (206 and 195) for a different, less potent seropositive sample (IR45270) when the same RBD-Alpha content (25 ng/mL RBD-biotin or 0.5 μM RBD-Alpha liposomes) was used (Figure 3D). However, the latter produced higher fluorescence intensities, which could only be matched with nav-liposomes when increasing the RBD-Alpha-biotin concentration by a factor of 2 again to 50 ng/mL (Figure 3C), resulting in a lower IC50 value (119) (Figure 3D). As expected, the concentration of RBD-biotin was a key factor in achieving good detection ranges, but it is inversely associated with the capture efficiency in the ACE2 plate. In the end, since the lower signal intensities enabled reliable detection, 25 ng/mL RBD-biotin was added to all nav-liposome assays to obtain good limits of detection. Furthermore, fluorescence-enhancing effects of serum constituents had to be taken into account for data processing, as signals of a negative control in the buffer were lower than those obtained for confirmed seronegative sera. This is exemplified by data normalization to the buffer control vs the lowest serum concentration for RBD-BQ1.1-biotin (Figure S12). The addition of HSA instead of serum had the same effect (Figure S13) and could be developed into a generic negative control, but natural variation of human serum will need to be assessed in the future for fine-tuning this negative control.

Finally, liposome concentrations of 1 μM total lipids and RBD-biotin concentrations of 25 ng/mL were chosen for the final assay, as they produced the highest signal intensities with the lowest amount of RBD, which corresponds to optimum sensitivity. The current assay format allows the separate storage of stav/nav-liposomes and RBD-biotin variants. In addition to the previously mentioned high storage stability of stav-liposomes at 4 °C, RBD-biotin was also shown to have excellent stability under the same storage conditions for several weeks. Alternatively, the conjugate can be stored, but additional stability studies are recommended. However, since naturally occurring biotin in patient samples may interfere with the assay format, preincubation of liposomes and RBD-biotin is recommended. Here, it was shown that when preincubating nav-liposomes and RBD-biotin, no interferences, i.e., false-positive signals, were observed with 575 nM free biotin (Figure S14), which resembles the threshold of the CLSI EP37 guideline^57^ for a 1:25 dilution of serum. This high biotin concentration was chosen as it resembles three times the highest concentration measured in a patient with high biotin dose uptake.

### Serum Panel Screening

Screening of five separate seronegative, prepandemic sera (four of them 1:1 pooled samples) showed some variations in nonspecific binding inhibition (Figure S15). The results were used to determine the cutoff values, which were calculated for each variant separately as the average binding inhibition values of all serum samples plus three times the standard deviation and ranged from 29% to 38% (Table S4).

Finally, 10 seropositive samples with different immunization and infection backgrounds (pre-Omicron exposure vs Omicron exposure, both groups including vaccine breakthrough infections) were tested for all five variants (Figure S16 and Table S5). All samples were correctly identified as seropositive. Furthermore, IC50 values could be obtained when the serum dilution was within the dynamic range of the assay. Specifically, reliable IC50 values could be obtained for 5 sera (S1, S2, S3, S6, S7) using a serum dilution range of 1:25 to 1:3200. For three sera (S4, S5, S10) with higher antibody titers, the serum dilution range was increased to 1:51200. In the case of sera S8 and S9, the antibody titers were outside of the tested range (IC50 > 3200). Based on this limited study, it is suggested to analyze patient sera starting with a 1:20 dilution followed by 2.5-fold series dilution, instead of the previously used 1:25 dilution followed by 2-fold series dilution.

Furthermore, the liposome assay could indeed provide relevant information regarding the neutralization potential of various virus variants. Grouping the sera based on the vaccination and infection history of the donors showed that sera from donors without Omicron booster or infection are overall less potent compared to Omicron-specific sera (Figure 4). Sera S1, S2, S3, and S7 show only very limited capability to neutralize Omicron variants but are able to neutralize Alpha and Delta with respectable titers. Serum S6 illustrates the immune escape of the Omicron, starting with a drop in IC50 values for BA.2/BA.5 compared to Alpha/Delta and another drop for BQ1.1. The same trend is visible for sera S4, S5, and S10, whose donors were exposed to Omicron, but had overall higher titers for all variants. S10 showed higher IC50 values for BA.2/BA.5 compared to Alpha/Delta, unlike the others. Overall, these data suggest that an Omicron vaccine breakthrough infection coincides with an increased neutralizing antibody breadth, with a bias toward higher titers against variants that are antigenically close to the initial immunization antigen, in line with previously published data.^31^ Furthermore, S8 and S9 illustrate that this imprinted bias might be, at least for a short time frame, overcome by repeated exposure to Omicron antigens, resulting in maximum neutralization breadth across all tested variants. These findings are also reflected in the antibody landscapes for the pVNT as well as the liposome surrogate neutralization assay, both of which reveal distinct patterns for Omicron vs no Omicron antigen exposure (Figure S17).

**Figure 4:**
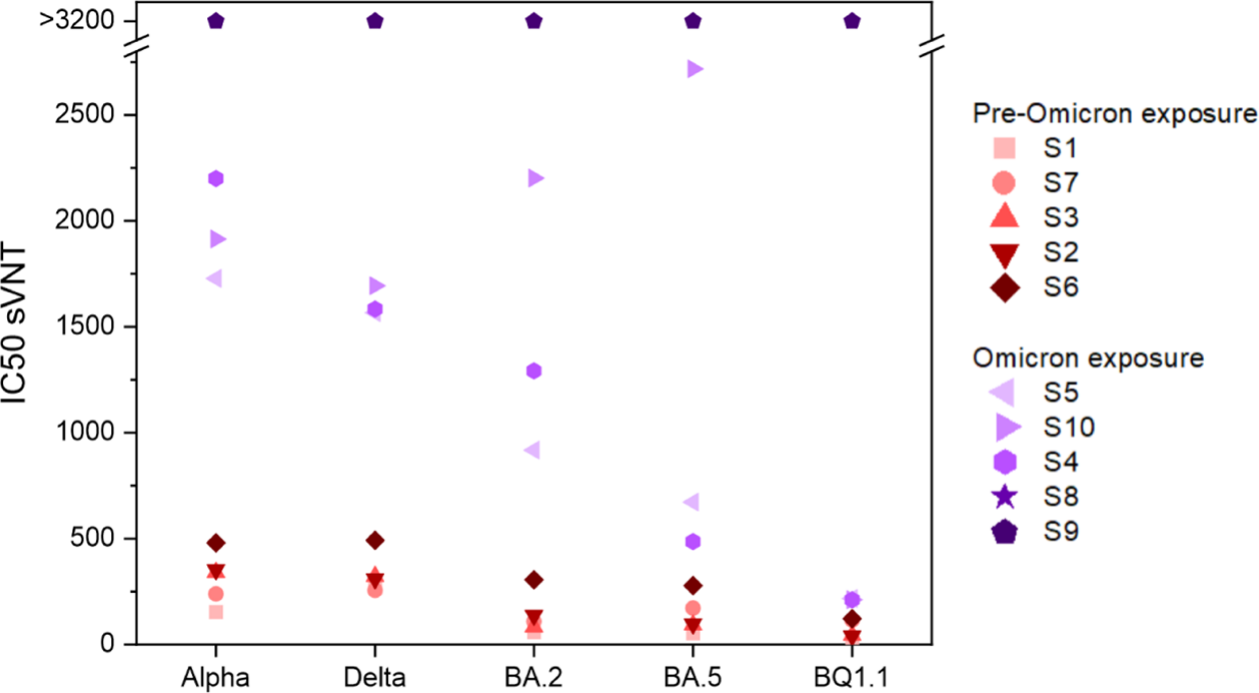
Comparison of IC50 values obtained with the 10 seropositive samples for each RBD variant in the liposome-based sVNT, sorted by IC50 values for Alpha and grouped into samples excluding and including the Omicron vaccine or infection. *n* = 3.

The liposome-based neutralization test results correlated excellently and were highly significant with those of the pVNT (Table S6), for both overall comparison (Spearman *R* = 0.86, *p* < 0.0001) as well as variant-specific comparison (all *R* > 0.9, all *p* < 0.00024) (Figure 5). These results show even higher correlations than traditional ELISA-based ACE2-binding inhibition,^54^ highlighting the potential benefit of a more virus-resembling liposome format. Overall, these findings demonstrate that liposome-based ACE2 inhibition assays represent a safer, scalable, and effective alternative to traditional virus-based neutralization tests.

**Figure 5:**
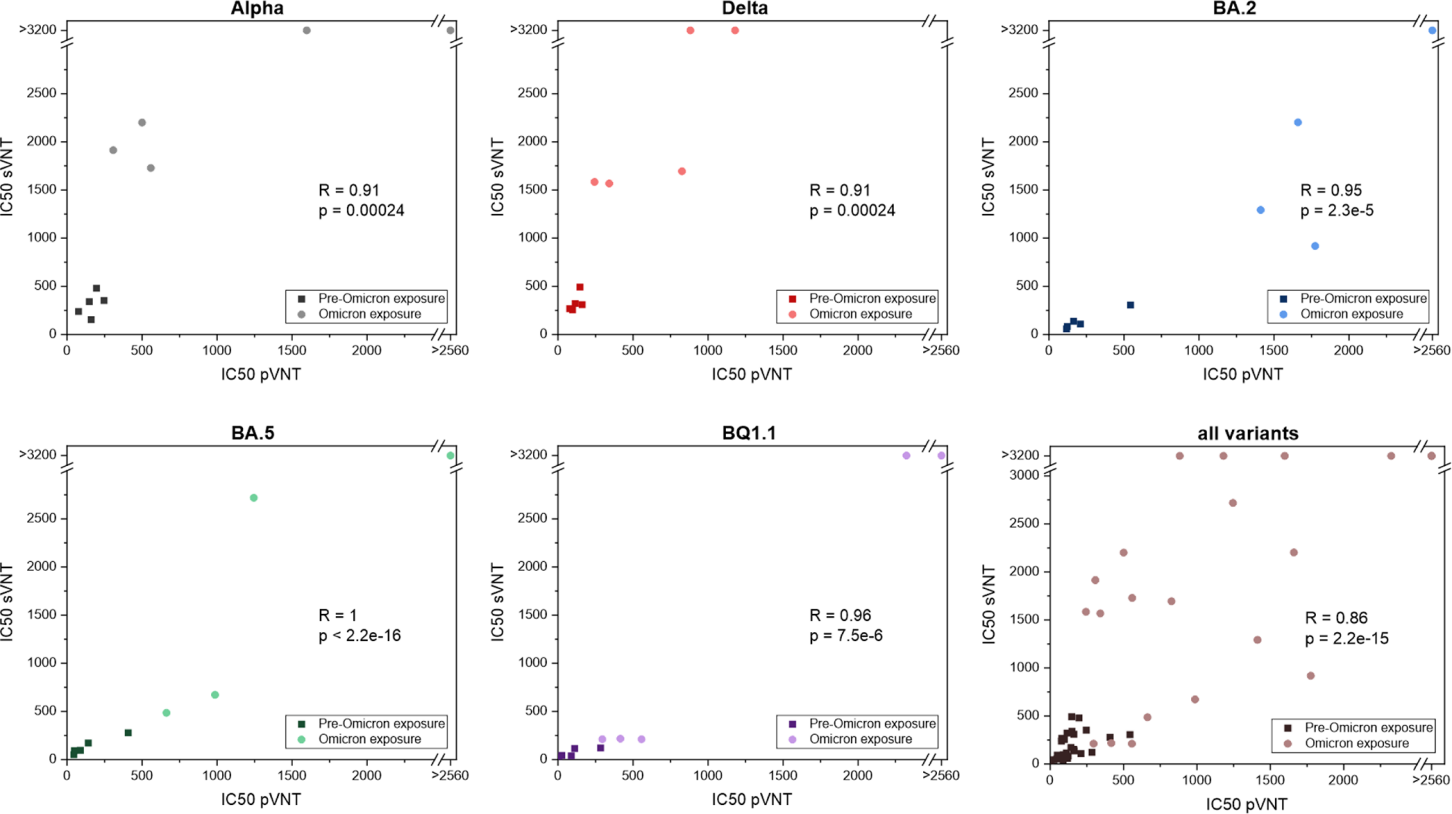
Spearman correlation of IC50 values obtained in sVNT and pVNT.

## Conclusion

Coupling of proteins to signaling labels is well established and is ubiquitously used. By using five variants of the SARS-CoV-2 RBD protein, we demonstrated how subtle sequence variations can lead to dramatically different coupling outcomes that are not easily predictable. Since the ultimate goal was the development of a standardized liposome-based assay platform technology, not only the protein coupling yield but also their functionality in binding to antibodies and the ACE2 receptor was carefully taken into consideration. Accordingly, altered physicochemical properties mediated by minor amino acid sequence variations can lead to an unfavorable orientation of proteins on the liposomal surface after direct EDC/sulfo-NHS coupling. This obstacle could be overcome only to a minor extent by random NHS-biotin-mediated coupling, suggesting that the proper orientation of RBD on the liposome surface rather than the distance to the liposome surface is crucial for favorable receptor interaction.

Site-specific biotinylation can mediate the proper orientation of the protein upon immobilization on streptavidin-liposomes. Strategies require genetic engineering of recombinant proteins and include the introduction of N- or *C*-terminal Avi- or Cys-tags for enzymatic or maleimide-mediated site-specific biotinylation and, at the same time, the avoidance of chemical alteration of lysine residues. Using RBD variants derived from 5 different VOCs, we highlight the advantages of proper orientation and topologically correct display of antigens on the liposome surface, thus supporting standardized and optimal interaction with the ACE2 receptor. Altogether, our results show excellent correlations with conventional virus neutralization, highlighting the potential benefit of a virus-resembling liposome format.

Looking into the future, click chemistry might be a promising approach to facilitate site-directed protein modification of liposomes without relying on the biotin–streptavidin interaction. Display of alkyne or azide residues on liposomes can be easily achieved by the addition of functionalized lipids during preparation or even via postinsertion. For the proteins, however, click chemistry requires the introduction of alkyne- or azide-residue-containing noncanonical amino acids,^16^ making it less straightforward than biotinylation of Avi- or Cys-tags.

## Supplementary Material



## Notes

Additional information regarding protein characteristics, figures and tables providing a more in-depth overview of the conducted assay optimization for EDC/sulfo-NHS coupling and the biotinylation strategies, as well as detailed results for the serum panel screening (PDF)
