# Supplementary material for: Development of a Liposome-Based Serological Assay for SARS-CoV‑2 Variants with Special Emphasis on Coupling Chemistries Required to Maintain Protein Antigenicity
Source: Anal Chem. 2025 Sep 5;97(36):19532–43. doi: 10.1021/acs.analchem.5c02526 (PMC12444751; doi:10.1021/acs.analchem.5c02526)
Supplement: Supplementary file 1 [file ac5c02526_si_001.pdf]

Supporting Information

Development of a serological liposome-based assay for SARS-CoV-2 variants with special emphasis on coupling chemistries required to maintain protein antigenicity

Simon Streif[1†], Patrick Neckermann[2†], Kilian Hoecherl[1], Christina Reiner[1], Sebastian Einhauser[2], Johannes Konrad[3], Miriam Breunig[3], Ralf Wagner[2,4*], Antje J. Baeumner[1*]

1 Institute of Analytical Chemistry, Chemo- and Biosensors, University of Regensburg, Universitaetsstr. 31, 93053 Regensburg, Germany

2 Institute of Medical Microbiology & Hygiene, Molecular Microbiology (Virology), University of Regensburg, Universitaetsstr. 31, 93053 Regensburg, Germany

3 Department of Pharmaceutical Technology, University of Regensburg, Universitaetsstr. 31, 93053 Regensburg, Germany

4 Institute of Clinical Microbiology and Hygiene, University Hospital Regensburg, Franz-Josef-Strauss-Allee 11, 93053 Regensburg, Germany

***** antje.baeumner@ur.de

***** ralf.wagner@ukr.de

[†] **contributed equally**

Table of contents

Protein characteristics 2

EDC/sulfo-NHS coupling 2

Biotinylation strategies for RBD 8

Establishing the surrogate virus neutralization test 11

Serum panel screening 16

References 20

Protein characteristics

Table S1: Theoretical molecular weight (MW) and isoelectric point (pI) of the five used RBD variants as calculated using protparam[1].

RBD variant	MW / Da	pI
Alpha	27023.27	8.11
Delta	27043.31	8.72
BA.2	27137.56	8.72
BA.5	27104.49	8.72
BQ1.1	27036.41	8.56

EDC/sulfo-NHS coupling

Modification of COOH-liposomes with RBD variants using EDC/sulfo-NHS chemistry led to a significant increase of the Z-average (Figure S1 A and Table S2) for Alpha, Delta and BA.2 and of the ζ-potential (Figure S1 B) for Alpha, Delta, BA.2 and BQ1.1 (one-way ANOVA, $p < 0.001$). In the case of BA.5 the Z-average remained at 152 nm, and the ζ-potential at -28 ± 2 mV compared to -32 ± 2 mV for unmodified liposomes, suggesting unsuccessful modification.

Assuming the liposomes to be monodisperse, unilamellar and spherical the theoretical number of RBD molecules per liposome was calculated to be 660 for an RBD mole fraction of 0.2 mol% using the following equation[2]:

$$N_{RBD\ per\ liposome} = N_{Lipid\ per\ liposome} * \chi_{RBD} = \left(\frac{\pi}{a_L}\right)[d^2 + (d - 2t)^2] * \chi_{RBD}$$

where d is the hydrodynamic diameter of the liposomes of 152 nm, t the bilayer thickness of 4 nm, and a_L the mean headgroup area calculated to be 43 Å², using the values of 19 Å², 71 Å², 45 Å² and 48 Å² for cholesterol, DPPC, DPPG and *N*-glutaryl-DPPE weighted with the respective mole fraction of 41.4 mol%, 32.2 mol%, 18.4 mol% and 8.0 mol% [3–5]. The encapsulant/lipid ratio was determined to be 0.26 mol SRB per mol lipid, resembling an encapsulation efficiency of 2.6%. The liposomes were estimated to contain ~85000 molecules of SRB with the above-mentioned assumptions, representing a ratio of 130 molecules SRB per RBD molecule.

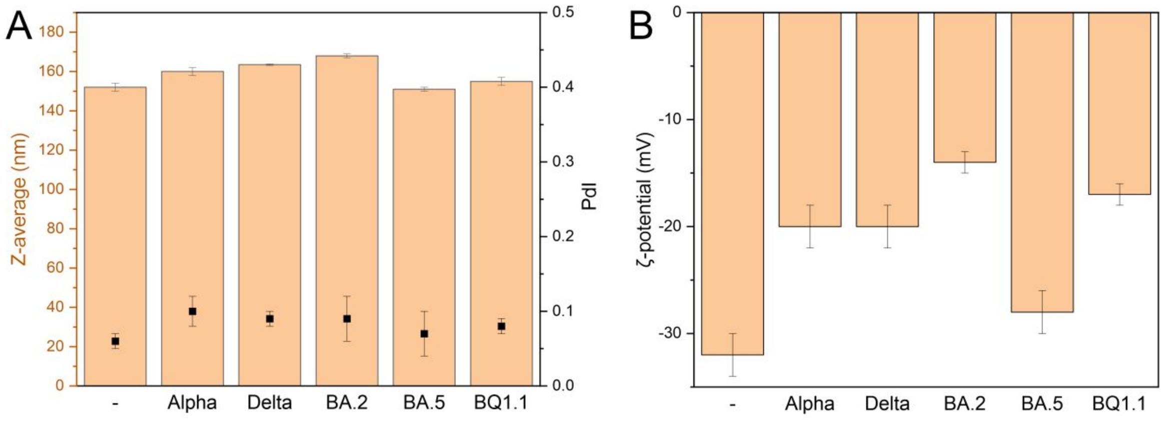

Figure S1: Z-average (A) and ζ-potential (B) of liposomes modified with 0.2 mol% of different RBD variants using EDC/sulfo-NHS chemistry. n = 3 (A) or 4 (B).

Table S2: Z-average and ζ-potential of liposomes modified with 0.2 mol% of different RBD variants or 1.38 mol% streptavidin or neutravidin using EDC/sulfo-NHS chemistry.

**Modification**	**Z-average / nm**	**PdI**	**Z-potential / mV**
-	152 ± 2	0.06 ± 0.01	-32 ± 2
0.2 mol% RBD-Alpha	160 ± 2	0.10 ± 0.02	-20 ± 2
0.2 mol% RBD-Delta	163.5 ± 0.4	0.09 ± 0.01	-20 ± 2
0.2 mol% RBD-BA.2	168 ± 1	0.09 ± 0.03	-14 ± 1
0.2 mol% RBD-BA.5	151 ± 1	0.07 ± 0.03	-28 ± 2
0.2 mol% RBD-BQ1.1	155 ± 2	0.08 ± 0.01	-17 ± 1
1.38 mol% streptavidin	156 ± 2	0.09 ± 0.01	-27 ± 2
1.38 mol% neutravidin	156 ± 2	0.10 ± 0.01	-22 ± 1

The sequence alignment of the RBD variants (Figure S2) revealed four mutations between Alpha and Delta, 16 between Delta and BA.2, three between BA.2 and BA.5 and three between BA.5 and BQ1.1 (Table S3). Several of these amino acids are responsible for the interaction with ACE2 (marked with grey arrows in Figure S2) or are neighboring ones that are (marked with red arrows). Besides affecting affinity to ACE2, the mutations have an influence on the orientation of RBD on the liposomal surface after EDC/sulfo-NHS modification, the sulfo-NHS-ester reacting with ε-amino groups of lysines.

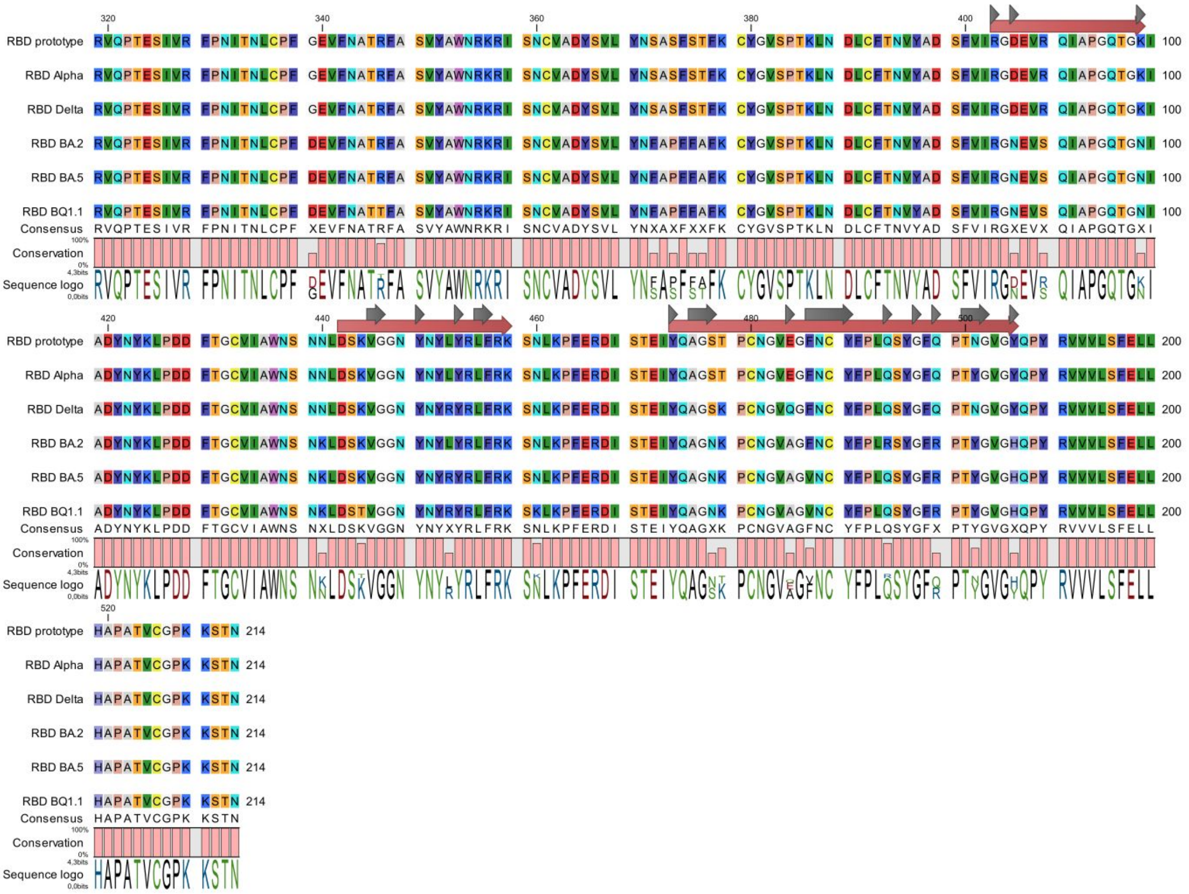

Figure S2: The sequence alignment of various RBD variants. Mutations of amino acids responsible for interaction with ACE2 are marked in grey. The red arrows mark the neighboring amino acids that could interact with said amino acids, influencing their interaction with ACE2.

Table S3: List of all mutations of RBD Alpha, RBD Delta, RBD BA.2, RBD BA.5 and RBD BQ1.1 in reference to the prototypic Spike nomenclature. Mutations of amino acids within the domains responsible for the interaction with ACE2 are marked with grey background, neighboring ones that could influence the interaction with light orange background and ones (theoretically) not affecting the interaction with white background. Nomenclature explained with the example G21D: amino acid G (glycine) in position 339 was exchanged with amino acid D (aspartic acid).

**Alpha**	**Delta**	**BA.2**	**BA.5**	**BQ1.1**
		G339D	G339D	G339D
				R346T
		S371F	S371F	S371F
		S373P	S373P	S373P
		S375F	S375F	S375F
		T376A	T376A	T376A
		D405N	D405N	D405N
		R408S	R408S	R408S
		K417N	K417N	K417N
		N440K	N440K	N440K
				K444T
	L452R		L452R	L452R
				N460K
		S477N	S477N	S477N
	T478K	T478K	T478K	T478K
	E484Q	E484A	E484A	E484A
			F486V	F486V
		Q493R		
		Q498R	Q498R	Q498R
Y501N		N501Y	N501Y	N501Y
		Y505H	Y505H	Y505H

NanoDSF measurements with a Prometheus Panta (NanoTemper Technologies GmbH) (0.36 mg/mL RBD in PBS, Prometheus standard capillaries PR-C002, initial measurement of the hydrodynamic diameter followed by thermal unfolding measurement from 20 °C to 90 °C with 0.05 °C intervals) revealed different melting points for RBD-BA.2 and -BA.5 compared to -Alpha, -Delta and -BQ1.1 (Figure S3 A). DLS measurements with the same device revealed that each variant is present in a different state of multimerization, hydrodynamic diameters ranging from ~4 nm for RBD-BQ1.1 to ~20 nm for -Delta. Denaturation during the thermal scan resulted in an increase of the hydrodynamic diameter for RBD-Alpha, -BA.5 and -BQ1.1 but not -Delta and -BA.2, which are present as larger multimers with ~12 nm and ~20 nm hydrodynamic diameters, respectively. It remains unclear whether this has an influence on coupling efficiency, because the coupling conditions, i.e., lower concentration, different buffer, and shaking could affect interactions between RBD molecules and thus multimerization.

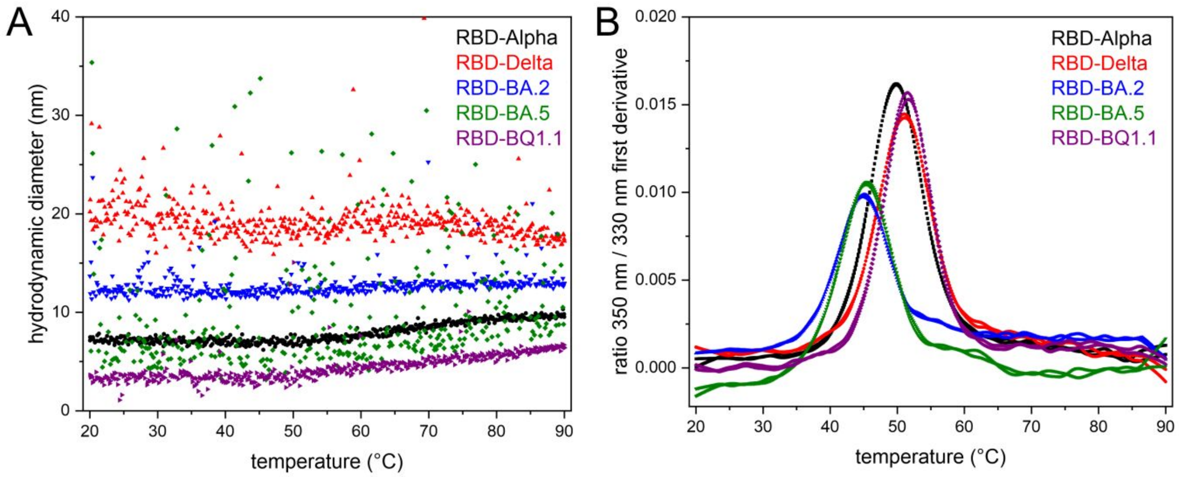

Figure S3: DLS measurement (A) and first derivative of the ratio of 350 nm to 330 nm fluorescence (B) during the thermal unfolding of RBD variants between 20 and 90 °C (0.05 °C intervals), measured in the Prometheus Panta. n = 2.

Binding of liposomes modified with 0.2 mol% RBD-Alpha using EDC/sulfo-NHS chemistry to ACE2-biotin immobilized in a streptavidin plate was analyzed over the course of 36 weeks (Figure S4 A). Modified liposomes (25 µM or 100 µM) were stored in PBS with additional 200 mM sucrose with and without HSA. HSA improved stability slightly for storage at 25 µM total lipids, though only decreasing the signal loss rather than preventing it. In the case of storage at 100 µM total lipids with HSA, liposomes maintained their ACE2 binding capacity for 24 rather than 8 weeks. However, after 36 weeks of storage they also produced >50% lower signal intensities. This could be in part due to degradation of RBD during storage, preventing binding to ACE2. Additionally, the liposomes continuously leaked encapsulant during storage, resulting in an increase of the unlysed fluorescence (Figure S4 B). Due to the high SRB concentration the fluorophore self-quenches when encapsulated in the liposomes. Once released the dilution in the outer buffer results in an increase of fluorescence. Leakage of SRB could not be prevented by an increase of the osmolality of the storage buffer by addition of sucrose or NaCl. Furthermore, no leakage was observed for the unmodified or streptravidin- or neutravidin-liposomes (data not shown), suggesting that the phenomenon is specific for RBD, which might interact with the lipid bilayer unfavorably.

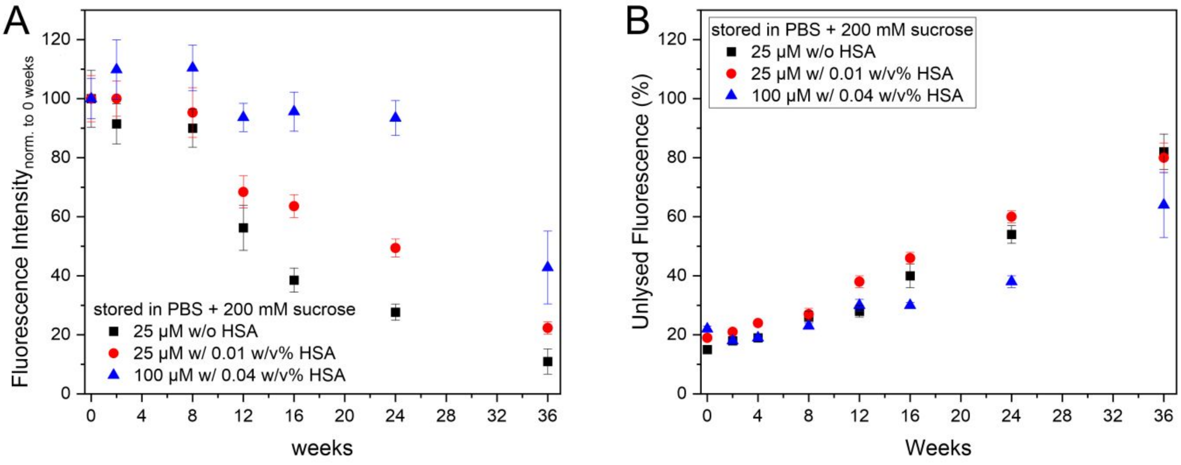

Figure S4: ACE2 binding (A) and unlysed fluorescence (B) of 0.2 mol% RBD-Alpha liposomes (stored at 25 or 100 µM in PBSS w/ and w/o HSA) after up to 36 weeks of storage, normalized to the initial intensities. A) Fluorescence intensities deviated significantly from the 0 weeks data-point for the 25 µM samples after 12 weeks, for the 100 µM sample after 36 weeks (one-way ANOVA, $p < 0.001$). $n = 3$.

Biotinylation strategies for RBD

Biotinylation of RBD-Alpha, -BA.5 and -BQ1.1 using 10 equivalents of NHS-biotin facilitated the capture of streptavidin-liposomes in an ACE2-coated high binding plate (Figure S5). Interestingly, even RBD-BA.5 facilitated capture, suggesting that the orientation upon biotinylation of NH_2-groups is preferrential to direct coupling to sulfo-NHS-activated COOH-liposomes via the NH_2-groups, possibly due to electrostatic repulsion. The use of 100 equivalents NHS-biotin per RBD resulted in excessive biotinylation, preventing interaction with ACE2 for all three variants. The use of lower equivalents of NHS-biotin was exemplarily investigated using RBD-Alpha (Figure S6). Highest signal intensities were obtained with 75 ng/mL RBD-Alpha-biotin modified using 5 equivalents NHS-biotin, and 100 ng/mL RBD-Alpha-biotin modified using 2 equivalents. Lower intensities were obtained for the conjugates biotinylated with 1 or 10 equivalents NHS-biotin, likely due to incomplete or excessive biotinylation.

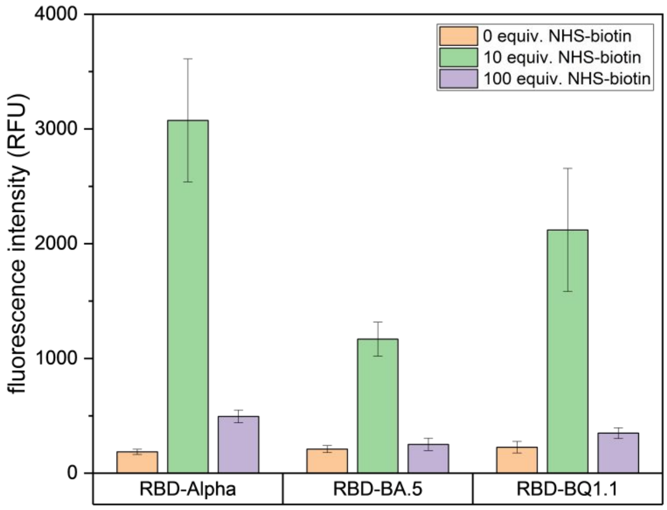

Figure S5: ACE2 binding of streptavidin-liposomes plus RBD-Alpha-, -BA.2- and -BQ1.1-biotin modified using 1, 10 or 100 equiv. NHS-biotin (150 ng/mL). n = 3.

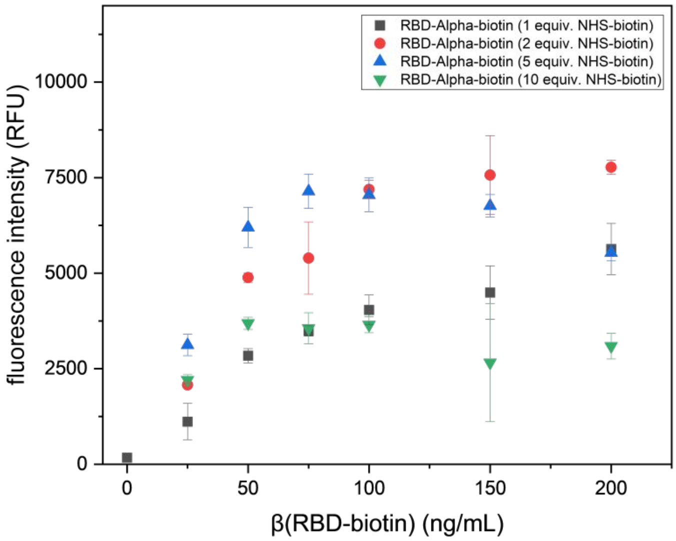

Figure S6: ACE2 binding of streptavidin-liposomes plus RBD-Alpha-biotin modified using 1, 2, 5 or 10 equiv. NHS-biotin (25 to 200 ng/mL). n = 3.

In order to control successful enzymatic biotinylation of the RBD proteins, a SDS-PAGE-based neutravidin shift assay was conducted (Figure S7). 50 pmol biotinylated RBD were incubated at 95 °C for 10 min in reducing SDS-PAGE buffer. After cooldown to RT, either 150 pmol neutravidin or PBS were added and incubated for 5 min. Biotinylated RBD/neutravidin complexes run at higher molecular weight in the SDS-PAGE compared to biotinylated RBD alone.

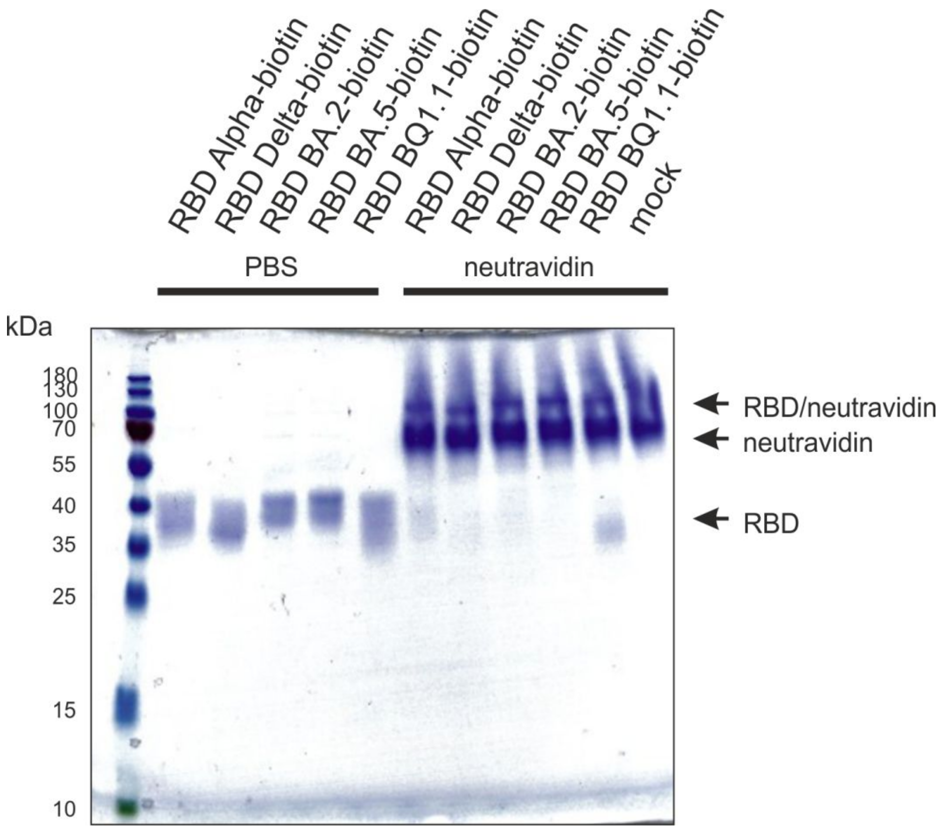

Figure S7: Neutravidin shift assay of enzymatically biotinalyted RBD proteins. Mock = PBS without RBD loaded.

Biotinylation via the Avi-tag using *E. coli* biotin ligase (BirA) facilitated the immobilization of streptavidin liposomes in an ACE2-coated high binding plate for all five RBD variants (Figure S8). Maximum signal intensities were obtained with 100 ng/mL RBD-biotin in all cases, though intensities varied between variants. Highest signals were obtained for Delta, which produced intensities comparable to those of RBD-Alpha-liposomes modified using EDC/sulfo-NHS chemistry. Alpha and BA.2 produced similar signal intensities, BA.5 and BQ1.1 produced the lowest intensities.

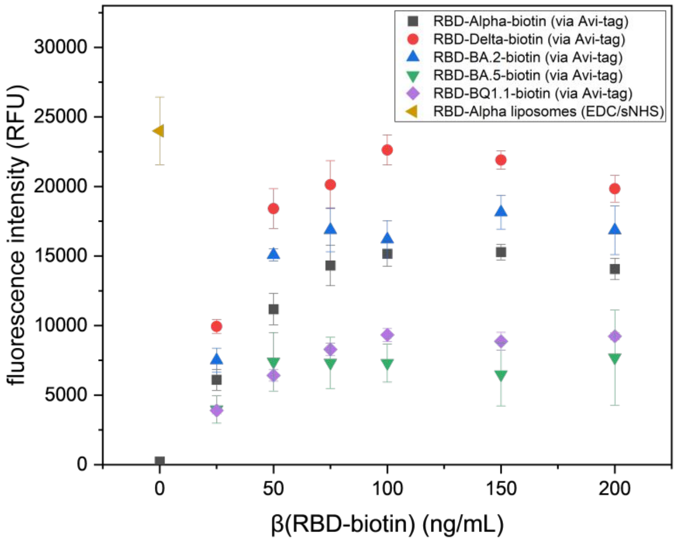

Figure S8: ACE2 binding of streptavidin-liposomes plus biotinylated RBD-Alpha, -Delta, -BA.2, -BA.5 and -BQ1.1 (modified via the Avi-tag) (25 to 200 ng/mL). n = 3.

RBD-BA.2-biotin resulted in extremely high non-specific binding of streptavidin-liposomes in a high binding plate blocked with BSA, coating of the plate with ACE2 did not result in a further signal increase (Figure S9). Blocking with skim milk powder (SMP) instead of BSA prevented non-specific binding and could be used for all variants.

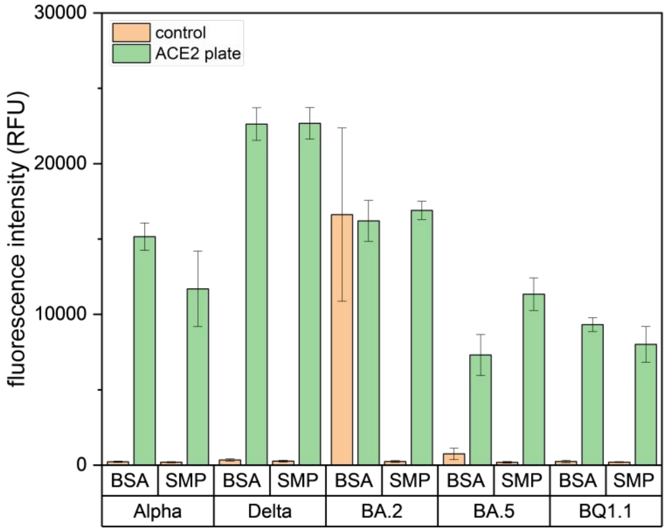

Figure S9: Fluorescence intensities of streptavidin-liposomes plus biotinylated RBD-Alpha, -Delta, -BA.2, -BA.5 and -BQ1.1 (modified via the Avi-tag) (100 ng/mL), immobilized in an ACE2-coated high binding plate blocked with skim milk powder (SMP) or BSA. n = 3.

Establishing the surrogate virus neutralization test

Pre-pandemic serum reduced signal intensities of RBD-Alpha-liposomes in an ACE2-coated high binding plate, correlating to increasing binding inhibition with increasing serum concentration (Figure S10). The avidity of the RBD-Alpha-liposomes to the randomly immobilized ACE2 appeared to be too low, serum constituents preventing their interaction. Site-directed immobilization of ACE2-biotin in a streptavidin plate improved avidity enough so that the seronegative sample did not show any influence on fluorescence intensities, corresponding to the expected binding inhibition values around 0%. The same was observed for streptavidin-liposomes with biotinylated RBD variants (data not shown). Therefore, ACE2-biotin coated and biotin blocked streptavidin-plates were used for the surrogate neutralization test using streptavidin-liposomes and biotinylated RBD.

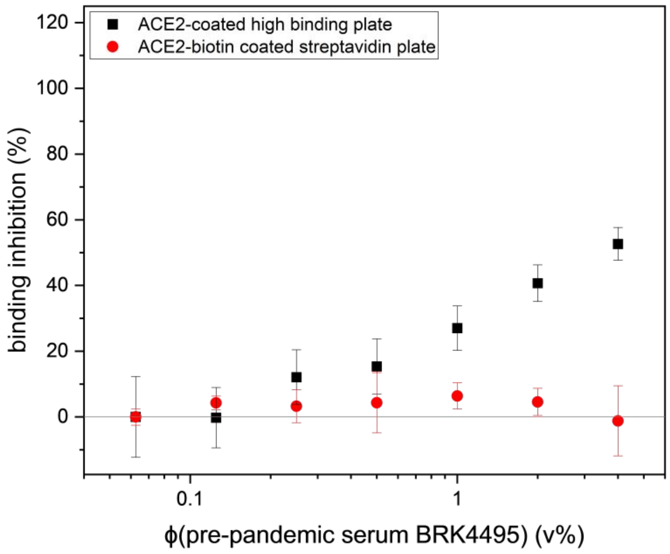

Figure S10: Binding inhibition values of RBD-Alpha-liposomes and pre-pandemic serum BRK4495, immobilized in an ACE2-biotin coated streptavidin plate or an ACE2-coated high binding plate. n = 3.

No non-specific binding of streptavidin- and neutravidin-liposomes was observed to an ACE2-biotin coated streptavidin-plate (Figure S11). The RGD sequence of ACE2 is inaccessible for binding of the RYD sequence of streptavidin. The same goes for the RGD sequence of RBD, probably due to binding beyond detection limit, as non-specific binding of streptavidin-liposomes to RBD would have led to the capture by immobilized ACE2. Thus, both streptavidin- and neutravidin-liposomes could be used for the SARS-CoV-2 surrogate virus neutralization test. Neutravidin-liposomes were chosen to make the assay more universally transferable to other analytes and matrices, which might contain proteins with accessible RGD sequence.

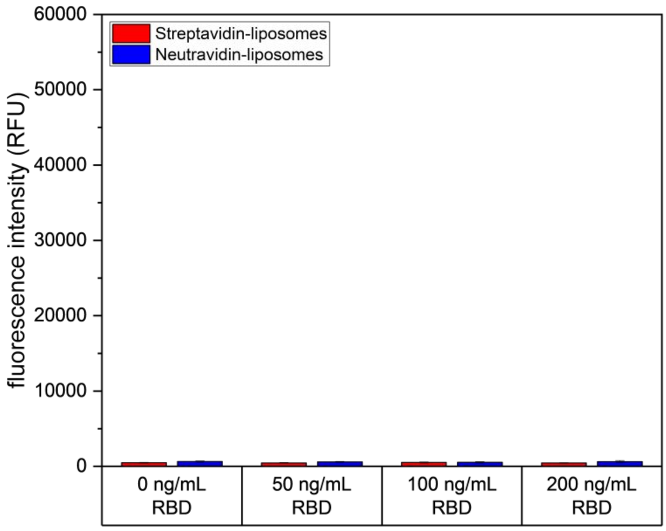

Figure S11: Fluorescence intensities of streptavidin- and neutravidin-liposomes incubated in an ACE2-biotin coated streptavidin plate (blocked with biotin) with RBD (0, 50, 100 or 200 ng/mL). n = 3.

Seronegative samples were found to increase the fluorescence intensity obtained with neutravidin-liposomes plus RBD-biotin in an ACE2-biotin coated streptavidin plate. The effect was most pronounced for RBD-BQ1.1-biotin, as it produced the overall lowest signal intensities. Investigation of varying neutravidin-liposome to RBD-BQ1.1-biotin ratios with pooled pre-pandemic serum showed the same effect for all conditions (Figure S12 A). Normalization to the serum-free controls thus led to negative binding inhibition values (Figure S12 B). Normalization to the highest serum dilution, on the other hand, resulted in the expected binding inhibition values around 0% (Figure S12 C). For the final studies a neutravidin-liposome concentration of 1 µM and an RBD-biotin concentration of 25 ng/mL was chosen. The lower signal intensities obtained with lower RBD-biotin concentration were deemed worth the trade-off as they improve sensitivity.

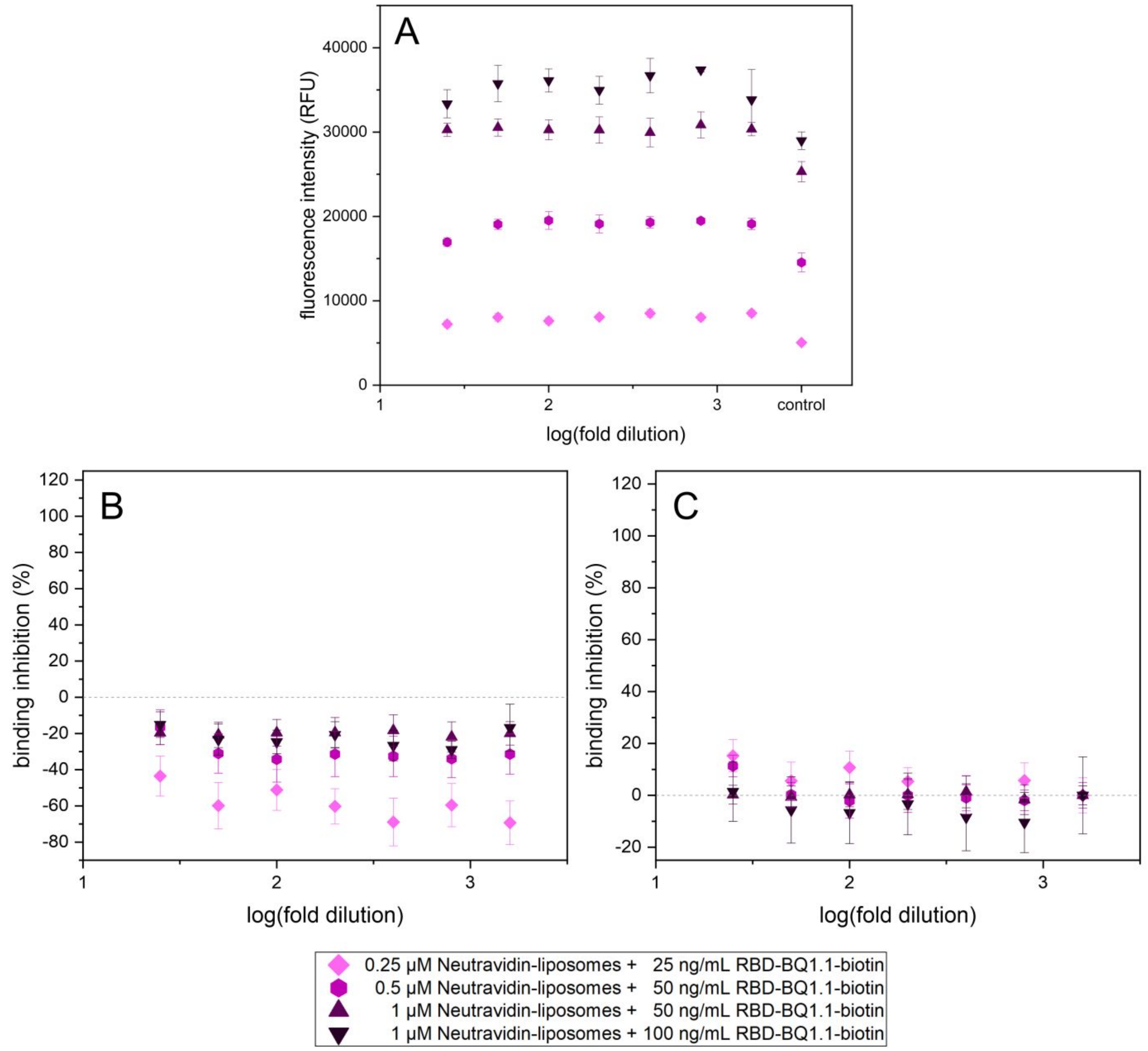

*Figure S12: Fluorescence intensities (**A**) and binding inhibition values (**B** and **C**) of neutravidin-liposomes (0.25, 0.5 or 1 µM total lipids) plus RBD-BQ1.1 biotinylated via the Avi-tag (25, 50 or 100 ng/mL) and pooled pre-pandemic serum (BRK-4507-8-pool). n = 3. Binding inhibition, given as percentage, was calculated by normalization to the serum free samples (**B**) or the highest serum dilution (**C**).*

Addition of HSA instead of serum also resulted in increased fluorescence intensities for neutravidin-liposomes plus RBD-BQ1.1-biotin in an ACE2-biotin coated streptavidin plate (Figure S13). Either interaction with HSA promotes capture of the liposomes in the plate, or it might reduce non-specific binding to the reaction vial during pre-incubation, despite the use of Protein LoBind Eppendorf Tubes. No improvement was observed with protein low binding tubes from a different supplier (Sarstedt SafeSeal Protein Low Binding reaction vial). While addition of HSA to the control might be feasible it would require detailed analysis of the average HSA concentration of sera. For the investigated serum normalization to the HSA containing control would result in positive binding inhibition values, which would bias the fit for IC50 calculation. Instead, normalization to the highest serum dilution was performed for the serum panel screening.

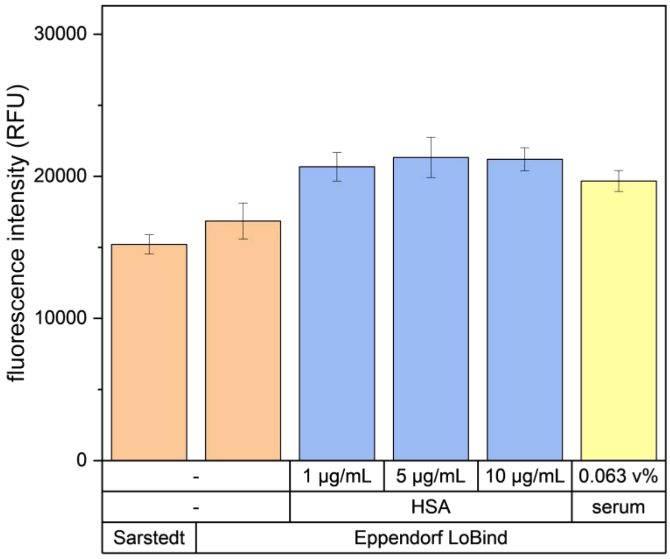

Figure S13: ACE2 binding of neutravidin-liposomes plus biotinylated RBD-BQ1.1 (modified via the Avi-tag) (50 ng/mL) in presence of HSA (0, 10, 50 or 100 µg/mL) or serum. Samples were pre-incubated for 1 h at 30 °C and 300 rpm in Sarstedt or Eppendorf low binding reaction tubes. n = 3.

Biotin interference was investigated by addition of 575 nM biotin instead of serum. This led to binding inhibition values below the determined cut-off values (see Table S4) for all five variants when neutravidin-liposomes and RBD-biotin variants are pre-incubated overnight before incubation with biotin (Figure S14). The biotin concentration corresponds to the biotin threshold according to the CLSI EP37 guideline, factoring in the 1:25 dilution of the highest serum concentration tested. Thus, no false-positives would be observed even for such excessively high biotin concentrations, corresponding to three times the highest measured value for a person with high biotin dose uptake. For a commercial product the instructions would state that no biotin uptake should have taken place within the last 24 h to ensure proper function of the assay.

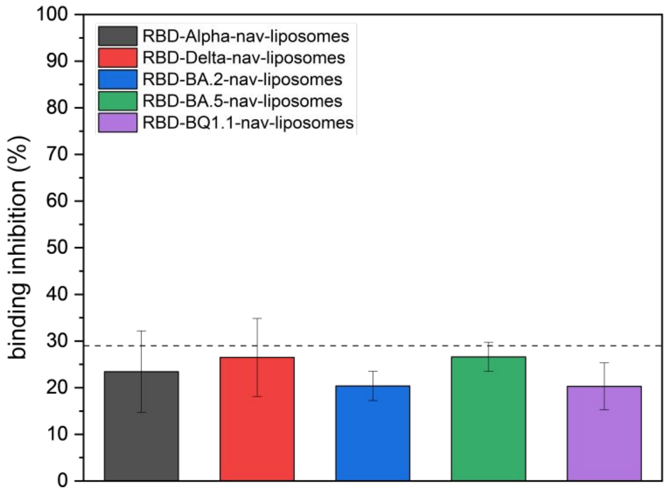

*Figure S14: Effect of biotin (575 nM) on the ACE2 binding of neutravidin-liposomes plus biotinylated RBD variants (modified via the Avi-tag) (25 ng/mL). Samples were pre-incubated overnight at 4 °C before incubation with biotin (0 or 575 nM) for 1 h at 30 °C and 300 rpm. n = 3. Binding inhibition, given as percentage, was calculated as (1 – fluor. int./fluor. int. 0 nM biotin)*100. The dotted line represents the lowest of the cut-off values determined using 5 seronegative samples (listed in Table S4).*

Serum panel screening

A total of five (pooled) pre-pandemic sera were tested for all five RBD-biotin variants. Obtained binding inhibition values showed variation (Figure S15). Cut-off values were calculated for each variant as the average of binding inhibition values obtained for all tested serum dilutions plus three times the standard deviation. Cut-off values ranged from 29% for Alpha to 38% for BQ1.1 (Table S4).

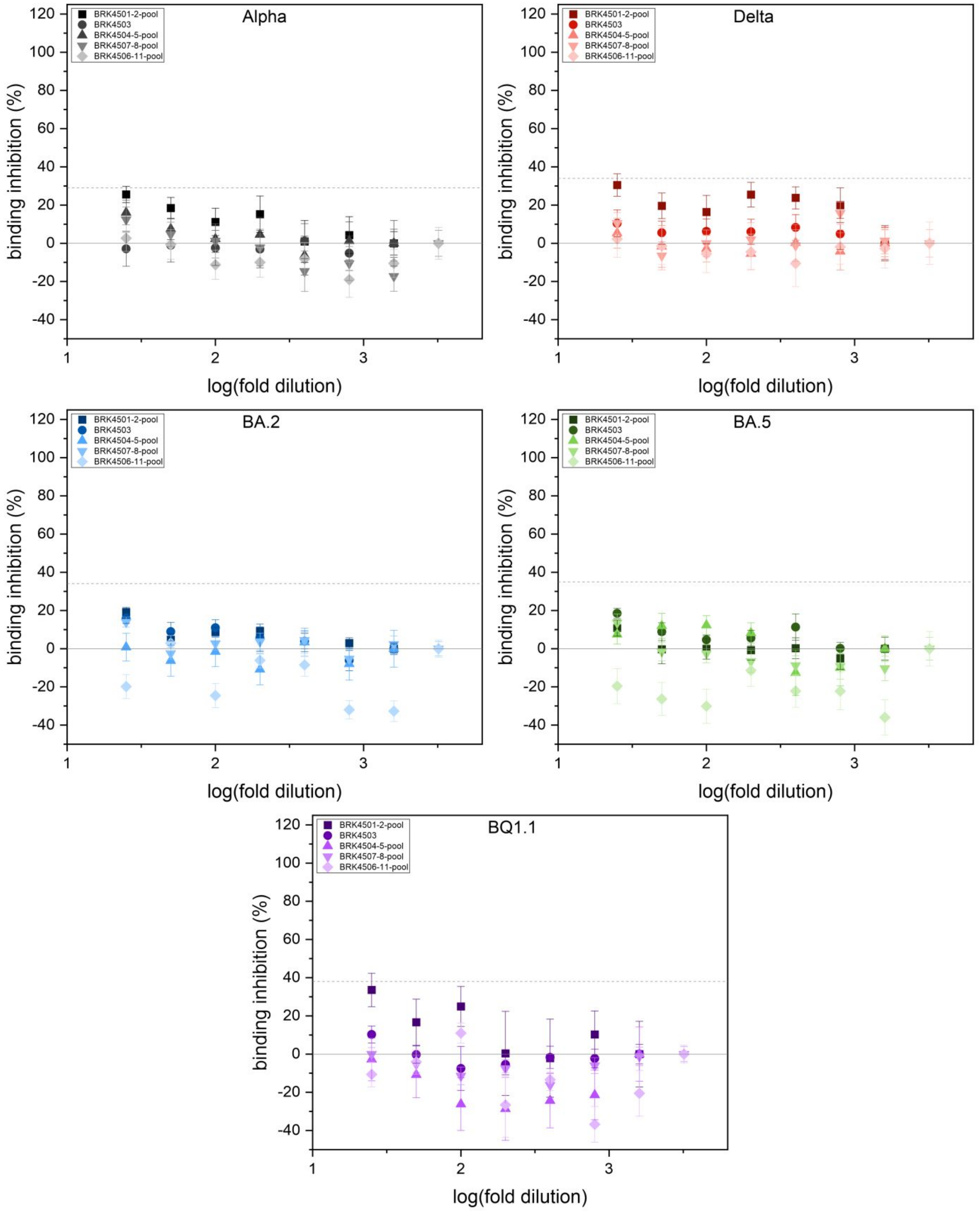

Figure S15: Screening of five seronegative samples with the neutravidin-liposome-based neutralization test for the different RBD variants biotinylated via the Avi-tag. n = 3.The cut-off value (dashed grey line) was calculated as the average plus 3x SD of all binding inhibition values obtained with the respective variant.

Table S4: Average binding inhibition values and standard deviations thereof for all five RBD variants. Cut-offs were calculated as the average plus three times the standard deviation.

Variant	Average binding inhibition	Cut-off (Average + 3 SD)
Alpha	0% ± 10%	29%
Delta	4% ± 10%	34%
BA.2	-1% ± 12%	34%
BA.5	-3% ± 13%	35%
BQ1.1	-5% ± 14%	38%

A total of 10 seropositive samples were screened for all five variants (Figure S16). Binding inhibition values above the cut-off values were obtained for all, enabling calculation of IC50 values (Table S5). One of these was below the investigated range, the highest concentration of serum S1 caused only 41% binding inhibition, the calculated IC50 value of 20 was hence classified as <25. In case of sera S8 and S9 all serum dilutions caused more than 50% binding inhibition for each variant, IC50 values were classified as >3200. Calculation of IC50 values for S4, S5 and S10 of Alpha, Delta, BA.2 and BA.5 required the testing of an additional four serum dilutions.

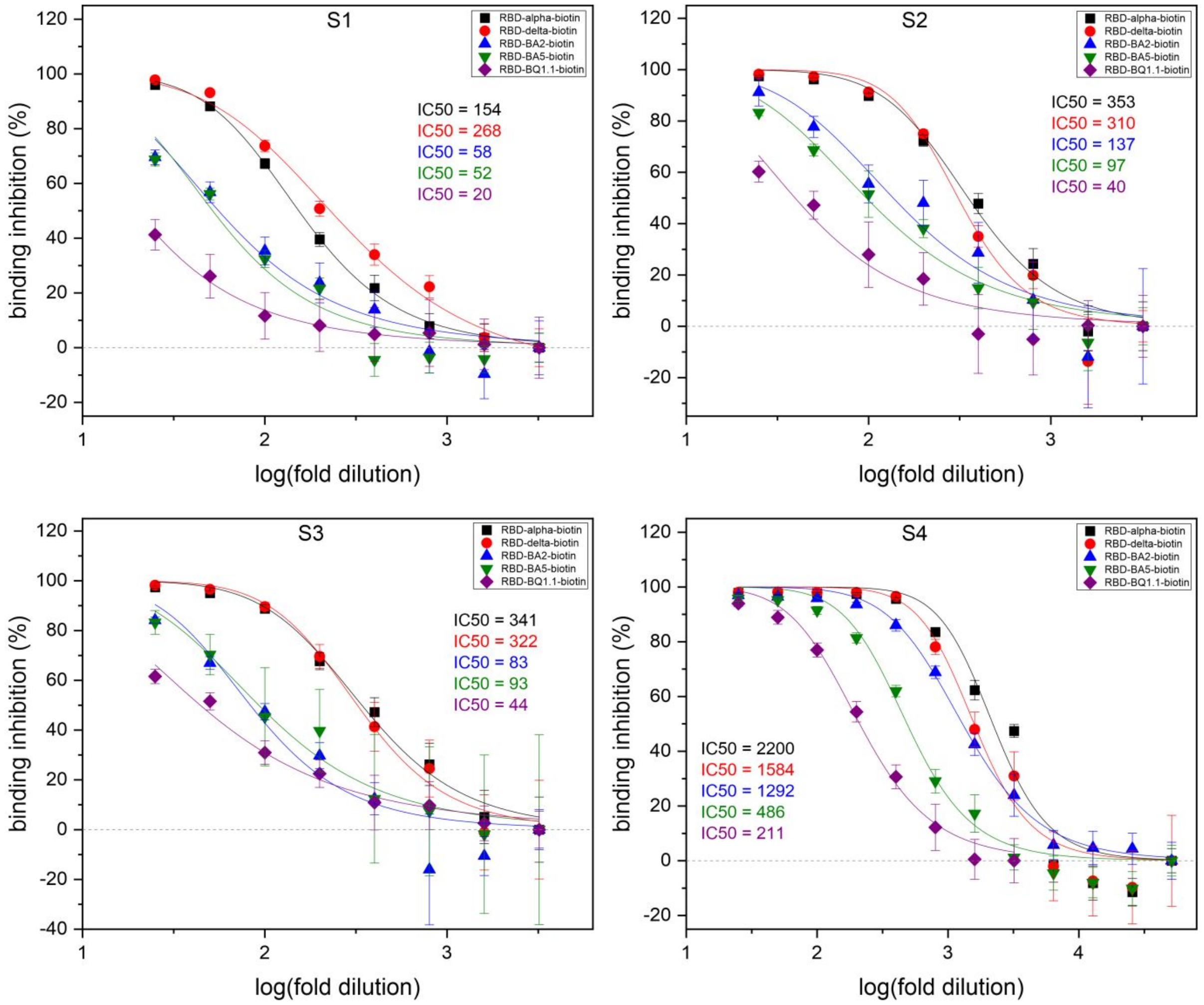

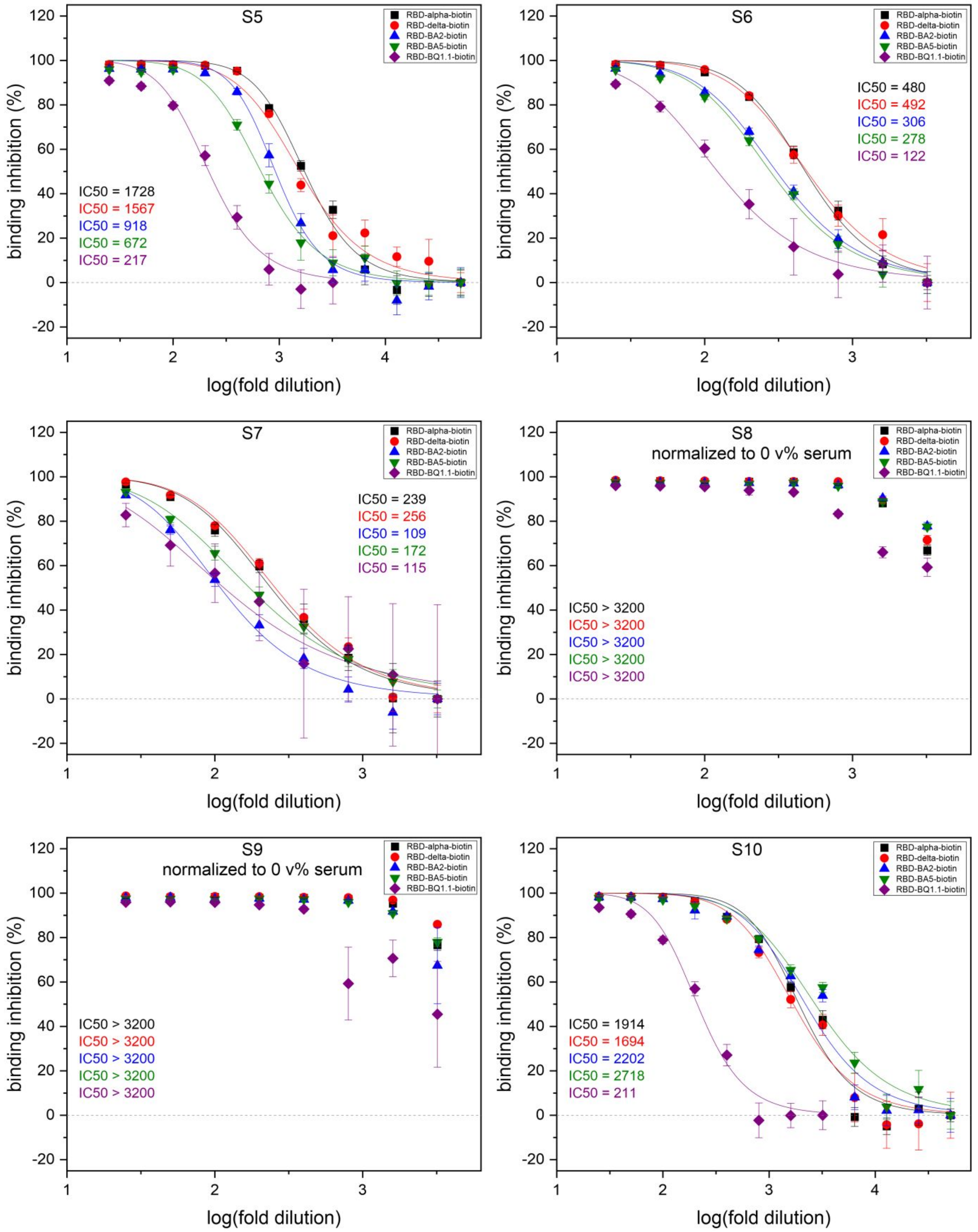

Figure S16: Screening of ten seropositive samples with the neutravidin-liposome-based neutralization test for the different RBD variants biotinylated via the Avi-tag. Binding inhibition was calculated by normalization to the highest serum dilution, unless stated otherwise. n = 3.

Table S5: sVNT IC50 values for Alpha, Delta, BA.2, BA.5 and BQ1.1 RBD obtained in the neutravidin-liposome-based neutralization test for the 10 seropositive samples. Sera from individuals without omicron antigen exposure are marked in light grey,sera with omicron exposure history are marked in light blue.

**Serum**	**Antigen exposure**	**alpha**	**delta**	**BA.2**	**BA.5**	**BQ1.1**
**S1**	WT vaccine	154	268	58	52	<25
**S2**	WT vaccine	353	310	137	97	40
**S3**	WT vaccine	341	322	83	93	44
**S4**	WT vaccine + Omicron exposure	2200	1584	1292	486	211
**S5**	WT vaccine + Omicron exposure	1728	1567	918	672	217
**S6**	WT vaccine + Delta exposure	480	492	306	278	122
**S7**	WT vaccine	239	256	109	172	115
**S8**	WT vaccine + Omicron exposure	>3200	>3200	>3200	>3200	>3200
**S9**	WT vaccine + Omicron exposure	>3200	>3200	>3200	>3200	>3200
**S10**	WT vaccine + Omicron exposure	1914	1694	2202	2718	211

Table S6: pVNT IC50 values for Alpha, Delta, BA.2, BA.5 and BQ1.1 RBD obtained in the pseudovirus neutralization test for the 10 seropositive samples. Sera from individuals without omicron antigen exposure are marked in light grey,sera with omicron exposure history are marked in light blue.

**Serum**	**Antigen exposure**	**alpha**	**delta**	**BA.2**	**BA.5**	**BQ1.1**
**S1**	WT vaccine	160.3	78.24	117.3	44.75	5.083
**S2**	WT vaccine	245.7	161	163.7	88.89	88.27
**S3**	WT vaccine	147.9	114.6	121.6	48.24	25.07
**S4**	WT vaccine + Omicron exposure	500.5	243.4	1412	662.7	556.8
**S5**	WT vaccine + Omicron exposure	558.6	341.5	1775	986.6	416.2
**S6**	WT vaccine + Delta exposure	195.9	146.1	543.2	407.2	284.1
**S7**	WT vaccine	76.22	96.29	209.2	141.5	110.3
**S8**	WT vaccine + Omicron exposure	>2560	1181	>2560	>2560	>2560
**S9**	WT vaccine + Omicron exposure	1598	882.9	>2560	>2560	2323
**S10**	WT vaccine + Omicron exposure	826.4	308.2	1660	1245	295.9

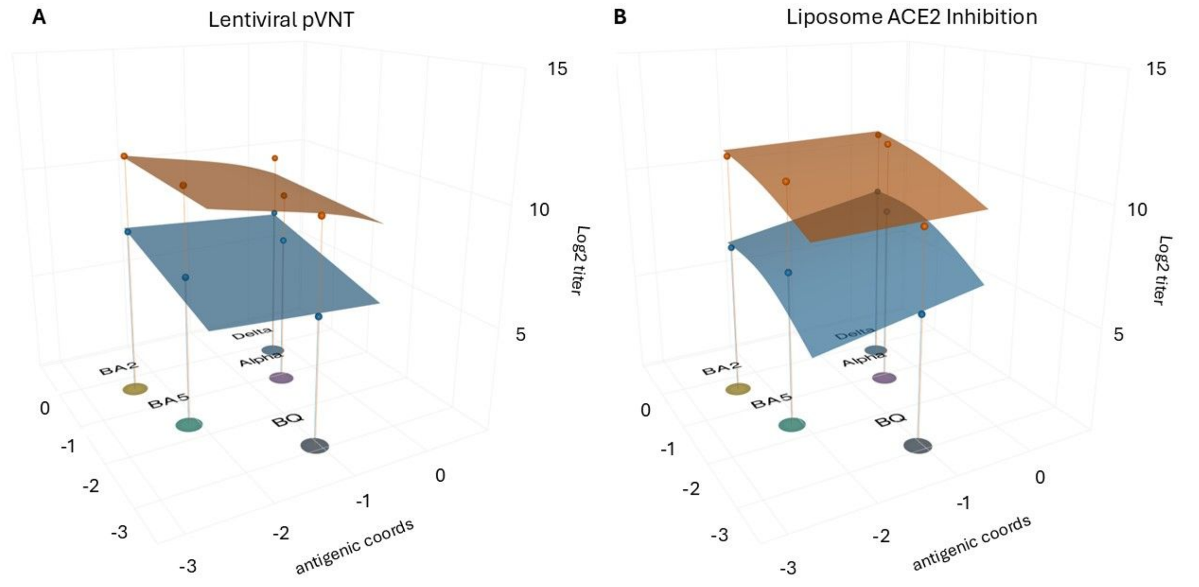

Figure S17: Antigenic Landscapes achieved for the lentiviral pseudotype neutralization assay (A) and the liposome surrogate neutralization test (B). Sera with no omicron antigen exposure are described in blue, with omicron antigen exposure after initial wildtype vaccination in orange.

References

(1) Walker, J. M. *The proteomics protocols handbook*; Humana Press, **2005**. DOI: 10.1385/1592598900.

(2) Fenzl, C.; Genslein, C.; Domonkos, C.; Edwards, K. A.; Hirsch, T.; Baeumner, A. J. Investigating non-specific binding to chemically engineered sensor surfaces using liposomes as models. *Analyst* **2016**, *141* (18), 5265–5273.

(3) Israelachvili, J. N.; Mitchell, D. J. A model for the packing of lipids in bilayer membranes. *Biochimica et biophysica acta* **1975**, *389* (1), 13–19.

(4) Ege, C.; Lee, K. Y. C. Insertion of Alzheimer's A beta 40 peptide into lipid monolayers. *Biophysical Journal* **2004**, *87* (3), 1732–1740.

(5) McQuaw, C. M.; Sostarecz, A. G.; Zheng, L.; Ewing, A. G.; Winograd, N. Lateral heterogeneity of dipalmitoylphosphatidylethanolamine-cholesterol Langmuir-Blodgett films investigated with imaging time-of-flight secondary ion mass spectrometry and atomic force microscopy. *Langmuir* **2005**, *21* (3), 807–813.
